# Key regulators control distinct transcriptional programmes in blood progenitor and mast cells

**DOI:** 10.1002/embj.201386825

**Published:** 2014-04-23

**Authors:** Fernando J Calero-Nieto, Felicia S Ng, Nicola K Wilson, Rebecca Hannah, Victoria Moignard, Ana I Leal-Cervantes, Isabel Jimenez-Madrid, Evangelia Diamanti, Lorenz Wernisch, Berthold Göttgens

**Affiliations:** 1 Department of Haematology, Wellcome Trust and MRC Cambridge Stem Cell Institute, Cambridge Institute for Medical Research, Cambridge UniversityCambridge, UK; 2 MRC Biostatistics Unit, Institute of Public HealthCambridge, UK

**Keywords:** gene regulation, haematopoiesis, mast cells, progenitors

## Abstract

Despite major advances in the generation of genome-wide binding maps, the mechanisms by which transcription factors (TFs) regulate cell type identity have remained largely obscure. Through comparative analysis of 10 key haematopoietic TFs in both mast cells and blood progenitors, we demonstrate that the largely cell type-specific binding profiles are not opportunistic, but instead contribute to cell type-specific transcriptional control, because (i) mathematical modelling of differential binding of shared TFs can explain differential gene expression, (ii) consensus binding sites are important for cell type-specific binding and (iii) knock-down of blood stem cell regulators in mast cells reveals mast cell-specific genes as direct targets. Finally, we show that the known mast cell regulators Mitf and c-fos likely contribute to the global reorganisation of TF binding profiles. Taken together therefore, our study elucidates how key regulatory TFs contribute to transcriptional programmes in several distinct mammalian cell types.

See also: **EH Davidson** (June 2014)

## Introduction

Transcription factor (TF) proteins decode the gene regulatory instructions encoded within our genomes and unsurprisingly therefore have emerged as key regulators of cellular identity. Individual TFs bind to short sequence motifs within gene regulatory regions, where the regulatory code is translated into gene expression changes through co-operative interactions between individual TFs as well as the recruitment of accessory proteins such as histone-modifying enzymes and the various components of the RNA polymerase holocomplex. TF binding to DNA therefore represents the first major information processing event during the regulation of gene expression and has therefore been a major focus of modern biomedical research ever since the first description of the lac operon (Jacob & Monod, 1961).

The advent of high-throughput sequencing technology greatly facilitated the generation of genome-wide TF binding maps, by directly sequencing the DNA fragments obtained via chromatin immunoprecipitation using the so-called ChIP-Seq technique (Johnson *et al*, 2007; Robertson *et al*, 2007). ChIP-Seq experiments not only form the backbone of several large international research initiatives (Adams *et al*, 2012; Dunham *et al*, 2012), but also represent a powerful tool for individual research laboratories. New insights generated using this approach range from the definition of new consensus binding motifs (Schmidt *et al*, 2012) and combinatorial TF interactions (Wilson *et al*, 2010) to previously unrecognised roles for repeat elements as substrates for gene regulatory evolution (Bourque *et al*, 2008).

Genome-wide TF binding maps, however, have so far delivered comparatively little progress in our understanding of TF-mediated control of cellular identity. Evidence has been provided for distal regulatory regions being critically important for the establishment of cell type identity (Heinz *et al*, 2010), and also the possibility that binding to promoters by at least some TFs can reinforce cellular identity (Rahl *et al*, 2010). Nevertheless, the sheer number of sites bound by a given TF, often more than 10,000, has made it difficult to gain a global understanding of TF-mediated control of cell type identity. The question has been raised therefore whether a large proportion of binding events are “opportunistic” rather than “functional” (John *et al*, 2011; Zhu *et al*, 2012), where “functional” would refer to those binding events that are relevant in terms of transcriptional control processes. Indeed, some high profile studies have restricted their analysis of TF binding sites to the minority of sites located in the vicinity of genes that change expression following external stimulation or TF knock-down (Garber *et al*, 2012).

Another important observation gained from ChIP-Seq studies concerns the realisation that global binding patterns for a given TF can be very distinct in different cell types (Wei *et al*, 2011) and indeed may correlate more strongly with other TFs in the same cell type than with itself across different cell types (Hannah *et al*, 2011). Of note, the cell type-specific nature of global binding patterns has also been observed for so-called master regulator TFs (Lodato *et al*, 2013), therefore raising the question as to how a “master regulator” can dictate cell type identity, when in fact it appears that its own global binding patterns are largely driven by the cellular environment? One possible explanation for this apparent paradox may of course be related to the notion of opportunistic binding, because if most binding events were to be opportunistic rather than functional, then the “opportunistic” binding events may be driven by the cellular environment, whereas the minority of “functional” binding events might relate to the cell identity conferring activity of a “master regulatory” TF.

The recent explosion in TF-mediated cellular reprogramming protocols (Takahashi & Yamanaka, 2006) only adds further urgency to research efforts aiming to close the very fundamental knowledge gaps in TF function outlined above, because a comprehensive understanding of how TFs interact with our genome will not only advance basic research but also be critical to gain a mechanistic understanding of cellular reprogramming strategies developed within the stem cell/regenerative medicine arena. Here, we report a comprehensive, genome-scale analysis of TF-mediated transcriptional control in blood progenitors and mast cells. Concerted mathematical and experimental analysis of global binding patterns for 10 key haematopoietic TFs in both cell types suggests that cell type-specific binding is not opportunistic, but instead makes meaningful contributions to cell type-specific transcriptional control. We furthermore illustrate how cell type-specific TFs likely contribute to the global reorganisation of TF binding profiles. Our findings therefore represent a relevant comprehensive computational and experimental analysis illustrating how multiple key regulatory TFs contribute to transcriptional programmes in distinct mammalian cell types.

## Results

### Many so-called blood stem cell “master regulators” are expressed similarly in mast cell and multipotent progenitors

We have previously established the multipotent and stem cell factor (SCF)-dependent HPC7 cell line (Pinto do *et al*, 2001) as a useful model for genome-scale analysis of transcriptional control mechanisms in early blood stem/progenitor cells (Wilson *et al*, 2009, 2010). In the absence of comprehensive data from other cell types however, it is impossible to perform the necessary comparative analysis required for the identification of those transcriptional mechanisms that contribute to cell type-specific control and cellular identity. Obtaining good quality TF ChIP-Seq data from blood cells can be challenging due to limiting cell numbers and population heterogeneity. Primary mast cells can be expanded as a homogenous population when bone marrow progenitors are cultured in the presence of IL-3 and SCF. To initiate comparative analysis, we generated RNA-Seq transcriptional profiles for both HPC7 and primary mast cells, with two biological replicates for each cell type. Each replicate produced approximately 30 million uniquely mappable reads, and expression values from biological replicates were highly correlated (Supplementary Fig S1).

**Figure 1:**
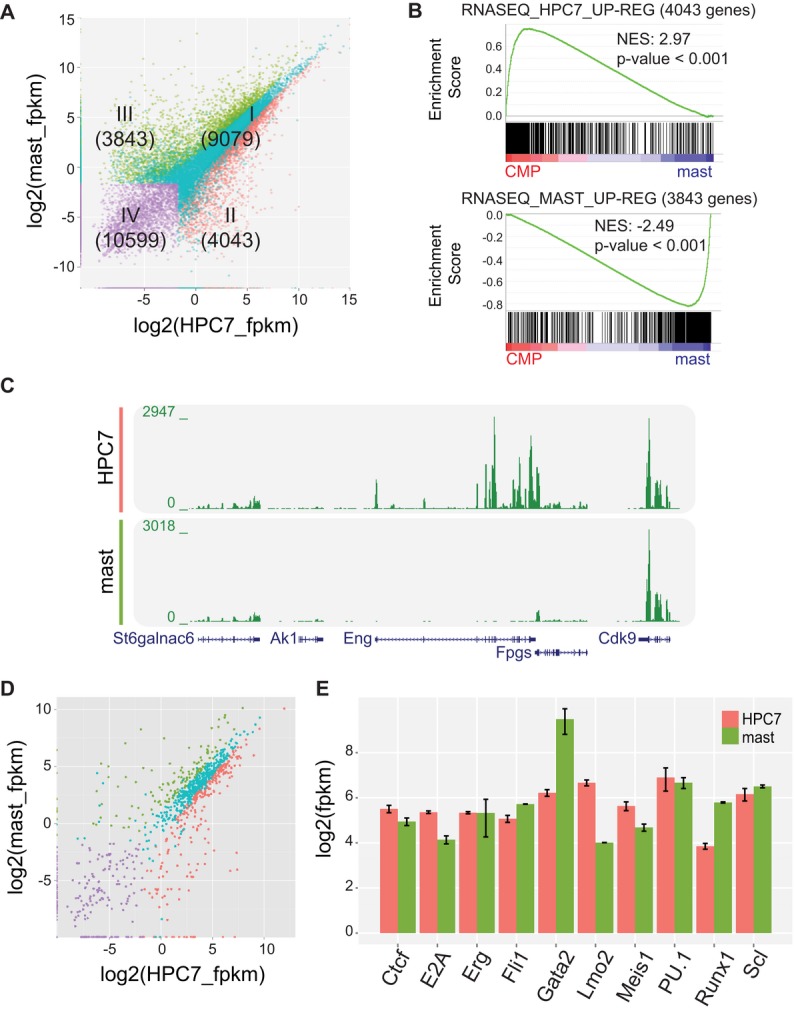
RNA-seq gene expression profiling of HPC7 and mast cells Scatterplot of *fpkm* values for all genes in both cell types. Each dot on the scatterplot is coloured based on 4 categories: (I) non-cell-type-specific, (II) HPC7-specific, (III) mast-specific and (IV) not expressed.Gene set enrichment analysis of genes in category (II) and (III) against the BioGPS expression dataset for CMP and mast cells. NES denotes normalised enrichment score.Genome browser screenshot of a gene expressed in haematopoietic progenitors (*Eng*) and a non-cell-type-specific gene (*Cdk9*). The *y*-axis represents read density along regions of known transcripts. Higher values correspond to more RNA transcripts.The same scatterplot as in (A) but only all known transcription factors are shown (data from RIKEN transcription factor database: http://genome.gsc.riken.jp/TFdb/). A large proportion of transcription factors are expressed at similar levels in HPC7 and mast cells, while a smaller subset are cell type-specific transcription factors.*fpkm* values of 10 key haematopoietic transcription factors.

Fig 1A shows the transcript expression levels in log_2_ *fpkm* values for all genes in HPC7 (*x*-axis) and mast cells (*y*-axis). Differential expression analysis revealed approximately 9K genes expressed at comparable levels in both cell types (category I), approximately 4K genes that are HPC7 or mast cell type-specific (categories II and III, respectively) and approximately 10K genes that are not expressed in either of the two cell types (category IV). Using independent microarray expression data for primary common myeloid progenitors and mast cells (Wu *et al*, 2009), we were able to demonstrate that genes specifically expressed in either HPC-7 or mast cells in our RNA-Seq data showed high enrichment in the primary CMPs and mast cells, respectively (Fig 1B). Of note, our HPC7 RNA-Seq gene set was more highly enriched in CMP expression data compared to other haematopoietic progenitors and other non-blood cell types (Supplementary Table S1). Together with visual inspection of individual gene loci (Fig 1C), GSEA allowed us to validate our expression data against previously established benchmarks.

Given our goal of comparative analysis of transcriptional control mechanisms, we next performed differential expression analysis for all known TFs (Fig 1D). Many TFs previously characterised as key regulators of HSC development and/or function were expressed at similar levels in both HPC7 and mast cells (see expression levels for *Scl/Tal1*, *Runx1*, *Erg*, *Meis1*, *Fli1*, *Pu.1* in Fig 1E). Some of these factors, like SCL/TAL1 (Salmon *et al*, 2007), GATA2 (Tsai & Orkin, 1997) and PU.1 (Walsh *et al*, 2002), had been implicated in mast cell function before, but no such information had been provided for the other HSC regulators. Similar levels of expression of key regulatory TFs suggested to us that, instead of quantitative variation between cell types, a distinct behaviour of TFs could contribute to specific transcriptional programmes.

### Genome-wide binding patterns are largely distinct for key regulatory TFs expressed in both HPC7 and mast cells

To perform a comparative analysis of genome-wide binding patterns of the key regulators simultaneously expressed in HPC7 and mast cells, we generated ChIP-Seq data for CTCF, E2A, ERG, FLI1, LMO2, MEIS1, PU.1, RUNX1 and SCL in primary mast cells and also CTCF and E2A in HPC7 cells. Together with our previously reported ChIP-Seq data for ERG, FLI1, GATA2, LMO2, MEIS1, PU.1, RUNX1 and SCL in HPC7 (Wilson *et al*, 2010) and GATA2 in mast cells (Moignard *et al*, 2013), this provided us with genome-scale data for the same 10 TFs in two cell types and would therefore allow us to investigate the contribution of shared TFs to cell type-specific transcriptional programmes.

**Figure 2:**
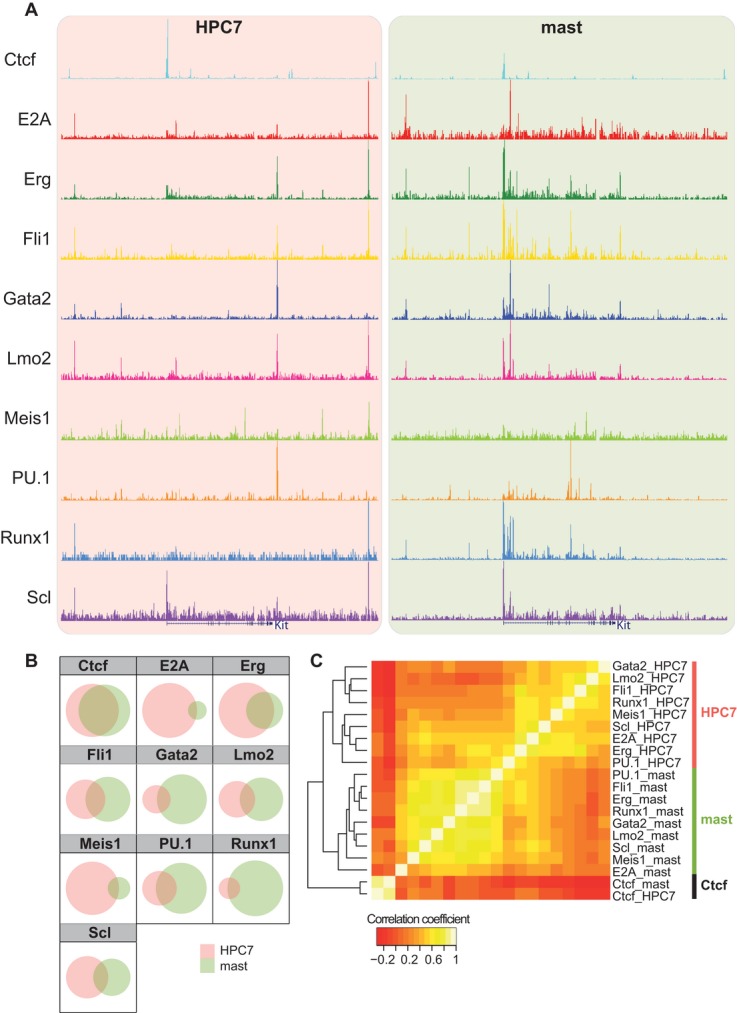
ChIP-Seq binding profile of 10 key haematopoietic transcription factors Comparison of binding sites on the *Kit* gene locus in HPC7 and mast cells shows many binding site differences between cell types.Global, binding site comparison between HPC7 and mast cells.Hierarchical clustering of the 20 global binding profiles. Each box in the heatmap corresponds to pairwise correlation coefficient of peak coverage data between pairs of samples in the row and column. Boxes on the diagonal indicate perfect correlation of a sample with itself. Ordering of samples in columns is identical to ordering in rows.

Visual inspection of ChIP-Seq results demonstrated very good signal-to-noise ratios for all 20 datasets and also suggested that binding profiles for most TFs diverged substantially between HPC7 and mast cells. This observation is illustrated in Fig 2A, which shows binding density profiles for all 10 TFs in both HPC7 and mast cells across the *Kit* gene locus. *Kit* is expressed at comparable levels in HPC7 and mast cells and encodes the receptor for SCF, a cytokine required for the growth of both HPC7 and mast cells. Comparison of the right (mast) and the left (HPC7) panels showed some overlap of binding peaks, but also substantial differences in binding locations for the same TF with several regions showing consistent binding by multiple TFs in either one or the other cell type. This observation suggested that even though the *Kit* locus is bound by all 10 factors in both cell types, the 10 TFs interact with the *Kit* gene locus in a cell type-specific manner.

To gauge the extent of cell type-specific binding at the level of the entire genome, we mapped binding peaks for all 10 TFs in both cell types and determined the extent of cell type-specific and shared peaks. This analysis demonstrated that with the exception of CTCF, all TFs showed largely non-overlapping binding sites (Fig 2B, Supplementary Table S2). Moreover, pairwise correlation analysis of all genome-wide binding profiles followed by hierarchical clustering demonstrated that with the exception of CTCF, binding patterns for the TFs clustered by cell type rather than the paired HPC7/mast cell datasets for the same TF (Fig 2C). These observations therefore indicate that the cellular environment can exert a major influence on global binding patterns where key regulatory TFs such as RUNX1, GATA2, MEIS1, SCL/TAL1 occupy largely non-overlapping parts of the genome in a cell type-specific manner within two closely related haematopoietic cell types.

### Genome-scale modelling reveals strong correlation between binding of shared TFs and cell type-specific gene expression

**Figure 3:**
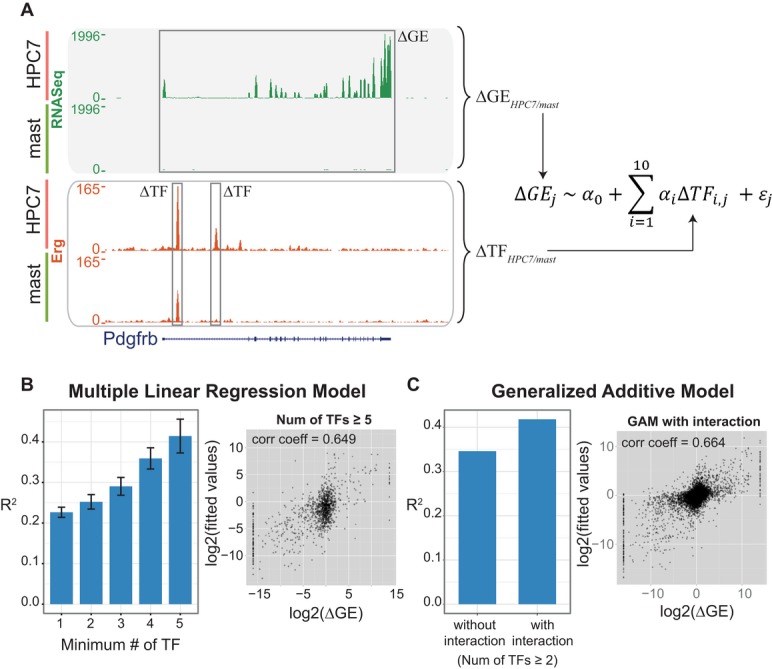
Mathematical modelling of gene expression and transcription factor variability The genome browser screenshot shows the *Pdgfrb* gene locus, a gene expressed in haematopoietic progenitors. This locus illustrates regions where differential scores were calculated. Differential transcription factor binding (ΔTF) was used as predictor variables in linear regression models to predict differential gene expression (ΔGE) as described in the equation on the right.Bar chart indicates the average *R*^2^ values with standard error from multiple linear regression models with tenfold cross-validation. *R*^2^ refers to the amount of variability explained by the model. Each model examines all genes bound by at least 1 up to 5 of the 10 shared TFs. 58.6% of genes expressed in either cell type are bound by at least 1 shared TF (Supplementary Fig S2A). The model with ≥ 5 transcription factors has the highest *R*^2^ value, which corresponds to a high correlation coefficient between observed and predicted values as shown in the scatterplot. Scatterplot corresponding to genes bound by at least 1 shared TF is shown in Supplementary Fig S2A.Average *R*^2^ values for generalised additive models on all genes bound by ≥ 2 transcription factors based on tenfold cross-validation. A higher *R*^2^ value was achieved using a GAM with interaction terms. The scatterplot shows the correlation between observed and predicted values in this model.

Having identified predominantly cell type-specific binding patterns for key regulatory TFs raised the question as to whether TFs are passively recruited to cell type-specific regions of open chromatin with no major regulatory impact, or whether they actively participate in two different transcriptional programmes. To evaluate the extent to which cell type-specific binding of shared TFs might be associated with gene expression, we developed multivariate linear regression models to correlate TF binding information in the two cell types as the predictor variables with gene expression data as the response variable (Fig 3A). Specifically, differential TF binding scores (ΔTF) for all shared TFs accounted for 10 predictor variables that were used to predict differential gene expression (ΔGE). TF-mediated control of gene expression was modelled taking into account both promoter and distal TF-bound regions.

Simple linear regression models including those genes bound by at least one TF (9,952 genes, Supplementary Fig S2A) showed some correlation between differential binding of shared TFs and gene expression in the two cell types (cross-validation *R*^2^ ˜ 0.227, Fig 3B), and scatterplots of predicted and observed values showed a moderate positive correlation (correlation coefficient ˜0.477, Supplementary Fig S2B). However, a significant group of genes with no change in gene expression was not predicted correctly (Supplementary Fig S2B). Since gene regulation often involves co-operativity between TFs, we next investigated model predictions using subsets of the original data by applying a minimum threshold for number of bound TFs. As shown in Fig 3B, increasing this threshold improved prediction accuracy reaching *R*^2^ values of 0.414 when genes bound by five or more TFs simultaneously were included (1,223 genes) and predicted values are much better correlated with observed values (correlation coefficient ˜0.649 for 5 or more TFs). A downside of this approach, however, is that predictions are only possible for a subset of genes if stringent cut-offs are implemented about the minimum numbers of TFs that must be bound to a given locus (Supplementary Tables S3 and S4).

We therefore sought to develop an alternative approach that would allow us to include co-operativity in our modelling without having to focus analysis on subsets of genes. In addition to the 10 predictor variables for 10 shared TFs, we examined the predictive power of 45 pairwise TF combination variables in a generalised additive model (GAM) (Hastie & Tibshirani, 1986; Wood, 2011). This approach uses smoothing functions of the predictor variables to fit single TF variables or concordant pairs of TFs to differential gene expression in a non-linear fashion. As shown in Fig 3C, GAM with interaction terms correlated more strongly with gene expression changes (adjusted *R*^2^ ˜0.417, correlation coefficient ˜0.645) than GAM without interaction terms (adjusted *R*^2^ ˜0.346, correlation coefficient ˜0.588) (Supplementary Table S5). We were also able to identify interesting TF pairs that co-operate to affect cell type-specific gene expression. For example, pairwise interaction between the ETS factors Erg and Fli1 with several other TFs was significantly correlated with gene expression changes (Supplementary Table S6).

Predictions obtained using GAM were more accurate than the corresponding results obtained from genes simultaneously bound by 2 or more TFs (8261 genes) with the linear regression model (cross-validation *R*^2^ ˜0.252) even in the absence of interaction terms. Importantly, our observation that differential binding of shared factors is predictive of differential gene expression is consistent with the notion that cell type-specific binding of shared TFs makes meaningful contributions to differential gene expression.

### Both cell type-specific and commonly bound regions show enrichment of consensus sequence motifs for the shared factors, but differ in their motif content for possible collaborating TFs

**Figure 4:**
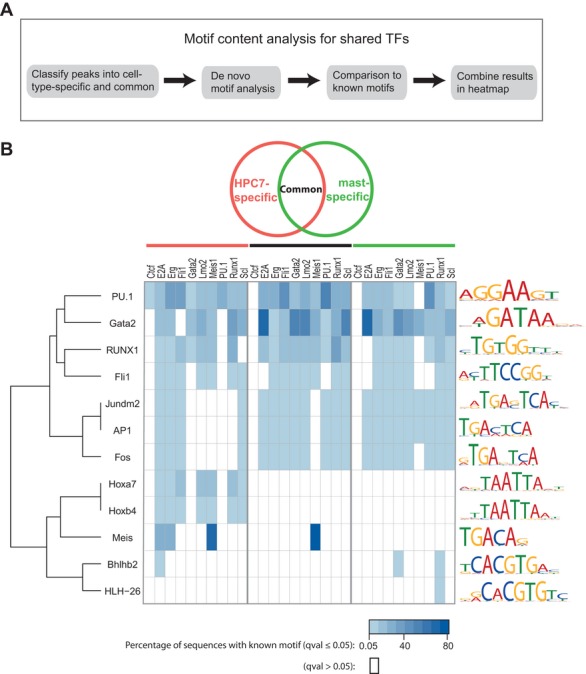
Motif content analysis of cell type-specific and common binding regions Method for conducting motif content analysis.Heatmap of a subset of all motifs tested. Blue rectangles denote significant (*q *≤ 0.05) similarity of enriched consensus sequence in a given dataset (column) to a known motif (row). See Supplementary Fig S3 for all motifs tested. Rectangles are coloured based on the percentage of regions that contained a given motif.

In view of our results, it remained unclear whether cell type-specific binding is largely mediated through tethering of shared factors to regulatory elements through protein-protein interaction with cell type-specific factors, or whether cell type-specific binding of shared TFs requires direct binding to DNA and, therefore, represents classical TF functionality. To investigate this question further, we carried out comprehensive motif analysis of common as well as cell type-specific TF-bound regions. Importantly, sequence motifs for key HSC regulators such as RUNX1, GATA2 and ERG/PU.1 were overrepresented across the board, that is, in HPC7-specific, shared and mast cell-specific TF-bound regions (see Fig 4). This observation therefore suggested that, just as for those regions bound in both cell types, direct DNA binding via established consensus motifs plays an important role for the binding of shared TFs to cell type-specific regions.

Having established that binding to cell type-specific peak regions appears to involve direct interaction with DNA, the question arises as to why some regions are only bound in one or the other cell type, and whether indeed cell type-specific TFs play a role in making these regions accessible for binding by the shared TFs. In the latter scenario, it should be possible to pinpoint any such consistent cell type-specific TFs through overrepresentation of their consensus binding sites within those regions bound by the shared TFs in only one or the other cell type. Indeed, we observed specific enrichment and/or depletion of consensus sequence motifs in the cell type-specific regions, such as an overrepresentation of Hox factor consensus binding sites in the HPC7-specific regions, depletion of AP-1 motifs in the HPC7-specific regions and also some specific occurrence of E-box motifs in the mast cell-specific regions (see Fig 4). This analysis therefore demonstrated that, in terms of their motif content, the HPC7-, common and mast cell-specific regions bound by the shared TFs are qualitatively different. For example, Hox motif overrepresentation in HPC7-specific regions was consistent with the known role for Hox factors in blood stem/progenitor cells and suggests that Hox factors may play a role in granting accessibility of shared TFs to HPC7-specific regions.

### Mitf and c-fos participate in binding of shared TFs to mast cell-specific regions

**Figure 5:**
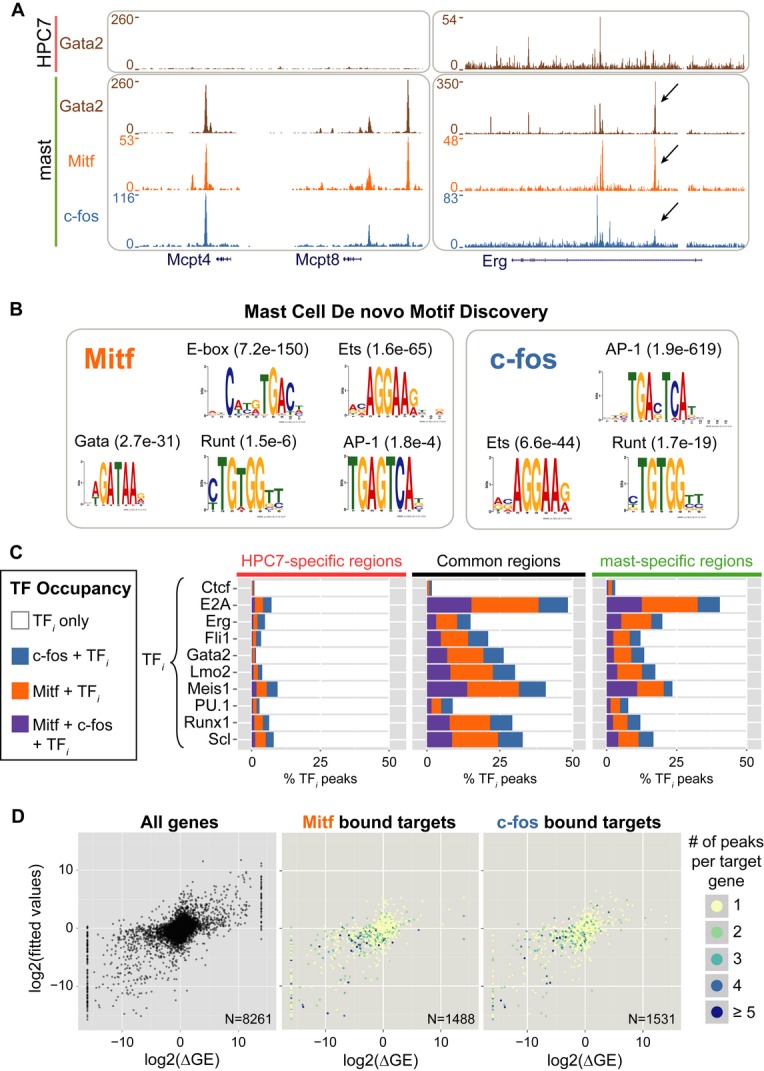
Mast cell-specific transcription factors, Mitf and c-fos Genome browser screenshots of the mast cell-specific gene loci *Mcpt4* and *Mcpt8* and a shared TF gene locus (*Erg*). On the left, mast-specific TFs and Gata2 bind to a new region in mast cells that is absent in HPC7. On the right, Gata2 binding is present in HPC7 but a new region in mast cells (arrowheads) is co-occupied with the mast cell-specific TFs.*De novo* motif analysis of Mitf and c-fos peaks.Co-occupancy between mast cell-specific and shared transcription factors in cell type-specific and common binding regions. For any given TF, TF_*i*_, the total number of peaks in each type of binding region (e.g. mast cell-specific) was taken as 100%.Observed versus predicted values scatterplot for the generalised additive model with pairwise TF interactions. From left to right, the scatterplot shows all genes, Mitf-bound genes and c-fos-bound genes, respectively.

Our analysis of DNA sequence motif content together with our RNA-Seq expression data allowed us to ask whether there are any TFs specifically upregulated in mast cells that are known to bind the mast cell-specific AP-1 and E-box motifs. Subsequent functional analysis would then allow us to assess their potential role in reorganising the binding profiles of shared TFs during the process of mast cell maturation. With respect to the AP-1 motif, mast cells expressed higher levels of *c-fos* and *c-jun* than HPC7, thus establishing *c-fos* as a candidate TF for mast cell-specific binding to AP-1 motif-containing regions. With respect to the E-box motif, the known mast cell regulator MITF similarly emerged as a candidate regulator and indeed was expressed over 47-fold higher in mast cells than in HPC7. To explore potential contributions of c-FOS and MITF to mast cell-specific binding of the shared TFs, ChIP-Seq experiments were performed for both c-FOS and MITF in primary mast cells. ChIP-Seq results showed that these 2 factors can be detected in mast cell-specific regions together with shared factors that were absent in HPC7 cells (Fig 5A, left panel). Motif analysis of binding peaks (Supplementary Table S7) revealed overrepresentation of the expected consensus binding sites for c-FOS and MITF as well as consensus motifs for some of the shared TFs such as GATA2, ERG/PU.1 and RUNX1 (see Fig 5B). These results therefore highlighted the possibility of coordinated binding by c-FOS and/or MITF with a subset of the shared TFs to mast cell-specific regulatory regions.

To directly investigate this hypothesis, we overlapped binding peaks of c-FOS and MITF with each of the shared TFs for common as well as cell type-specific regions. As shown in Fig 5C, c-FOS and MITF in mast cells co-occupy a substantial proportion of the regions bound by the shared TFs. MITF and c-FOS also bound to a substantial proportion of regions bound by shared TFs in both mast cells and HPC7, but not to HPC7-specific regions. These binding patterns are illustrated, for example, at the *Erg* gene locus, where c-FOS and MITF bind to regions together with shared TFs such as GATA2 and thus occupy regions in mast cells that are already bound in HPC7 cells (Fig 5A, right panel). In addition, MITF and c-FOS also bind to regions not bound in HPC7, and this “new” binding is accompanied by relocation of shared TFs such as GATA2 to these regions (see arrowhead in Fig 5A, right panel). Taken together therefore, these data are consistent with a model whereby mast cell-specific and shared TFs contribute to gene regulation in mast cells by binding to both shared and mast cell-specific regulatory regions. In order to assess the relative contribution of MITF and c-FOS to mast cell-specific expression, we overlapped the scatterplot comparing actual HPC7/mast cell expression differences with those predicted from our GAM, specifically for those genes that are bound by MITF or c-FOS (Fig 5D). The group of genes that were correctly predicted by the 10 TF model associated predominantly with single binding events for MITF and c-FOS within their gene locus. However, those genes that are expressed at higher levels in mast cells than predicted by our model associated strongly with multiple (> 2) binding events of MITF or c-FOS. Our results therefore indicate that cell type-specific factors such as MITF contribute to the global organisation of the shared TFs interrogated in this study and likely play important roles in cell type-specific transcriptional control beyond the gene loci that are differentially bound by shared TFs.

In order to investigate the effect that the addition of MITF and c-FOS would have to the predictive power of GAM, we then recalculated our model including these two factors (see Supplementary Fig S4 and Supplementary Tables S8 and S9). We may not have seen a significant improvement in *R*^2^ value when fitting 12 predictor variables because Mitf and c-FOS binding correlates with the 10 shared TFs. Nevertheless, the new model incorporates approximately 700 more genes, and thus, we were able to explain similar amounts of variation (as the 10TF model) but on a larger set of genes.

We wondered what known features of regulatory regions might correlate with the ability of our model to make good predictions. It has been suggested that acetylation of lysine 27 of histone H3 (H3AcK27) is a good mark to identify active regions of the genome (Creyghton *et al*, 2010). To investigate whether there is a link between this histone mark and our model predictions, we generated genome-wide profile for H3AcK27 in HPC7 and mast cells (Supplementary Fig S5). Interestingly, we observed that there is a strong association (chi-square test *P*-value ˜0) between those genes with high H3AcK27 in at least 1 cell type and genes that are well predicted (absolute residuals ≤ 1.5) by the GAM (≥ 2 TFs with interaction) (Supplementary Fig S6A). To exclude the possibility that this strong association was a contribution of non-differentially expressed genes, we performed the chi-square test on differentially expressed genes only and showed that the association is still significant (Supplementary Fig S6B). Taken together, our model captures biologically relevant links between differential TF binding and differential gene expression, which shows a significant overlap with other known features of active gene regulatory elements (such as H3AcK27) and with likely other features.

### Knock-down and functional promoter studies demonstrate active participation of HSC regulators in mast cell-specific transcriptional control

**Figure 6:**
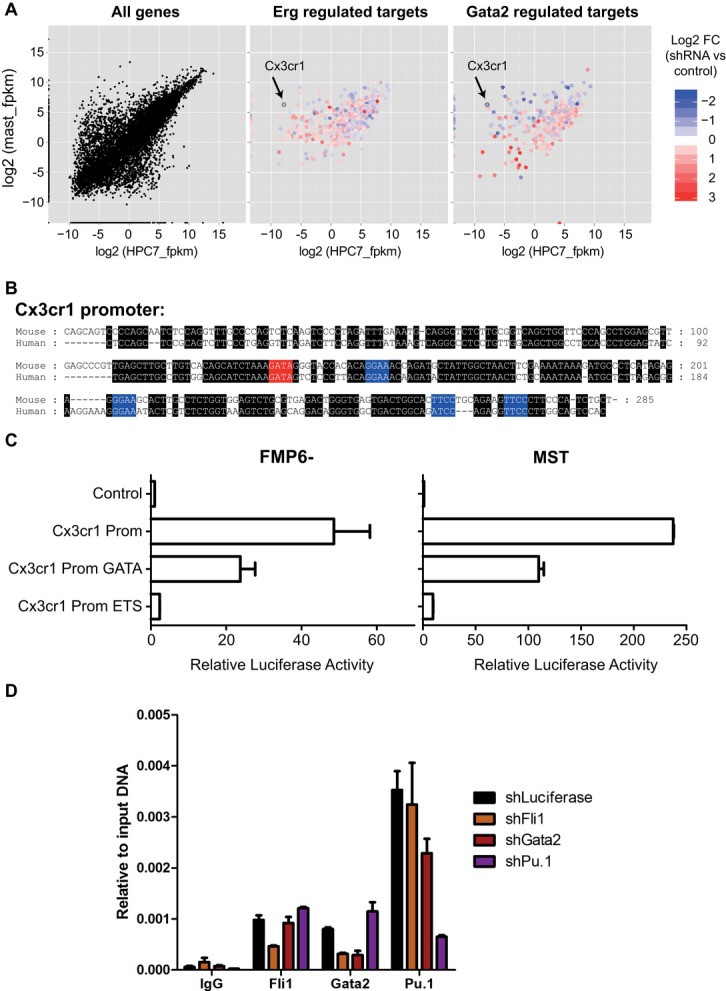
Perturbation of key haematopoietic TFs, Erg and Gata2 HPC7 versus mast scatterplot of RNA-seq *fpkm* values. From left to right, the scatterplot shows all genes, Erg-regulated targets and Gata2-regulated targets, respectively. Points on the scatterplot are coloured based on the log2 fold change of shErg or shGata2 compared to control. Only genes that are differentially expressed in the knock-down (absolute log FC > 0.38, *P*-value ≤ 0.05) are shown. The complete list of regulated targets and expression values can be found in Supplementary Table S10.Nucleotide sequence alignment of the *Cx3cr1* promoter region. Conserved GATA (red) and Ets (blue) motifs are highlighted.MST and FMP6- cells were electroporated with luciferase reporter constructs containing either the promoter or 2 different mutant versions of the promoter (GATA motif or simultaneous mutation of 4 conserved ETS motifs). Mean and SEM for two independent transfections (each one performed in triplicate) are shown. Values are expressed relative to the pGL2-Basic vector (Control).Real-time PCR analysis of ChIP for FLI1, GATA2 and PU.1 following knock-down of the same genes in the *Cx3cr1* promoter region. Experiments were performed in MST mast cell line and results are expressed relative to total DNA input.

While results in the previous section underscored the importance of cell type-specific factors such as MITF for cell type-specific expression, our mathematical modelling and DNA sequence motif content analysis also suggested that shared TFs play an active role in mast cell-specific gene expression. To evaluate the contribution of shared TFs to mast cell-specific expression experimentally, we performed knock-down experiments for several shared TFs in primary mast cells, followed by microarray gene expression profiling. TFs were knocked down following transfection of retroviral shRNA vectors with shRNA against luciferase serving as a negative control (Supplementary Fig S7A). The fraction of differentially expressed genes following TF knock-down with binding peaks for the respective TF in their loci ranged from 10.8 to 70.3% (see Fig 7EB). We next overlapped differentially expressed genes with binding peaks with our initial expression scatterplot (Fig 6A and Supplementary Fig S8) to exclude expression changes due to indirect effects. Interestingly, knock-down of shared TFs under analysis had a profound effect on the expression of mast cell-specific genes. In the case of *Gata2*, its reduction mainly caused downregulation of mast cell-specific genes, consistent with the known activating role of GATA2 (Masuda *et al*, 2007). In the case of *Erg* knock-down, the numbers of mast cell genes that were up- and downregulated were similar. To analyse the importance of shared TFs for mast cell growth, we also performed growth competition experiments in the mast cell line MST between uninfected cells and those where one of the shared TF was knocked down. Results showed that TF knock-down caused a growth disadvantage, with particularly striking results for *Fli1* and *Gata2* (Supplementary Fig S9).

To confirm that expression changes following shared TFs knock-down involved canonical motifs as predicted from our motif analysis, we next analysed the promoter of the *Cx3cr1* gene, which is expressed specifically in mast cells and is downregulated in primary mast cells following *Erg*, *Fli1* and *Gata2* knock-down (Supplementary Fig S10A). The *Cx3cr1* promoter was bound by ERG, FLI1, PU.1 and GATA2 (Supplementary Fig S10B) and also contained relevant binding sites conserved between mouse and human (Fig 6B). Luciferase assays showed that the *Cx3cr1* promoter is highly active in the mast cell lines FMP6- and MST (Fig 6C). Moreover, mutation of the single conserved GATA motif reduced activity more than 50% and simultaneous mutation of all 4 conserved ETS-family motifs abolished promoter activity. To analyse the possible effect of protein-protein interactions in protein recruitment, we performed knock-down experiments for *Fli1*, *Gata2* and *Pu.1* in the mast cell line MST followed by ChIP for the same factors, which allowed us to assess TF recruitment to the *Cx3cr1* promoter region by qPCR (Fig 6D). This analysis demonstrated that reduction in FLI1 levels reduced the recruitment of GATA2, yet recruitment of ETS proteins FLI1 and PU.1 was not affected by reduction in GATA2. Similar results were obtained in two additional regions (Supplementary Fig S11). Taken together, these results demonstrate that complex combinatorial interplay of shared TFs such as ERG, FLI1, PU.1 and GATA2 contributes to mast cell-specific transcriptional control.

## Discussion

Combinatorial TF interactions are critical determinants of cell type identity, a phenomenon particularly well understood within the blood system. Mast cells are a vital component of the immune system and also play a key role in multiple pathologies including allergic and autoimmune disorders, yet mast cells have remained one of the least understood blood lineages. Here, we show by comparative expression analysis that, unlike for erythroid, granulocyte/macrophage or lymphoid lineages, mast cells display comparable expression levels of a whole set of blood stem cell-affiliated TFs. Shared TF expression between HSPCs and mast cells may in part be consequence of a shared dependence on SCF signalling. Mast cells and HSCs are, however, functionally very different cell types. To investigate this apparent contradiction between co-expression of key regulators and different biological function, we performed a comprehensive genome-scale comparative analysis of gene expression and TF binding. In-depth experimental and computational analysis revealed: (i) largely non-overlapping binding profiles of shared TFs which are predictive of differential gene expression, (ii) cell type-specific TFs are likely drivers of global TF binding patterns of shared TFs and (iii) cell type-specific binding of shared TFs actively contributes to cell type-specific gene expression. Taken together therefore, our results argue against the currently prevalent model that a large proportion of TF binding events are opportunistic with no immediate transcriptional function. Of note, repeated cycles of moving between experimental and computational analysis were required to make a compelling case for this important point.

Contemporary biological research increasingly employs mathematical modelling approaches, not only because the size of datasets is growing exponentially, but also because the development of a mathematical framework consistent with biological observations offers potential insights into underlying mechanisms and permits *in silico* experimentation to select the most promising hypotheses for subsequent experimental validation. Previous attempts to model gene expression from TF binding data have largely focused on multi-TF ChIP-Seq data from a single cell type (Ouyang *et al*, 2009; Karlic *et al*, 2010; Marbach *et al*, 2012). More recent studies supported the idea that quantitative differences in TF binding signals between different cell types reflect the differences in gene expression but surprisingly using TF binding data from more than one cell type did not improve the performance of linear regression models (Cheng & Gerstein, 2012; Cheng *et al*, 2012; Handstad *et al*, 2012). In contrast, our implementation of GAM substantially improved the *R*^2^ values (0.252 versus 0.417). We would argue that GAMs are more representative of current transcription factor knowledge because they take into account non-linear and combinatorial transcription factor effects on gene expression. Moreover, our recursive strategy of moving between experimentation and computation prompted us to perform additional ChIP-Seq experiments for Mitf and c-fos, which in turn allowed us to revisit the GAMs in conjunction with the Mitf and c-fos datasets. This analysis showed that genes more strongly upregulated in mast cells than predicted by differential binding of the shared factors commonly contained several binding peaks for the mast cell-specific TFs. What might have been perceived as a deficiency in our model was therefore utilised to make new discoveries about transcriptional control mechanisms that may distinguish two closely related cell types.

High-throughput sequencing has transformed the analysis of transcription factor function allowing for the first time the production of genome-wide binding maps in complex mammalian genomes. The generation of new insights from these largely descriptive genome-wide maps has, however, proved much harder than might have been anticipated. One particular puzzling result was the observation that unlike with genes, where genome sequencing showed mammalian genomes to have far fewer genes than had been expected (Lander *et al*, 2001), TF binding events in many cases far exceeded expectations and commonly outnumber the total number of genes. A real disconnection has therefore opened up between the number of genes affected by TF perturbation such as knock-out or overexpression, which is commonly in the low hundreds, compared with the number of genes that have a TF binding peak in their locus, which is often in the middle to high thousands. One conclusion often drawn from this disparity is that a large proportion of TF binding events may be “opportunistic” rather than “functional,” where it is thought that TFs bind to regions that happen to be accessible and contain relevant motifs, without this binding being particularly relevant in terms of transcriptional control processes. While it is undoubtedly true that such opportunistic binding could represent a substrate for evolution and therefore serve some function, the notion that the majority of binding events may be non-functional presents major conceptual problems; for example, no clear sequence or other features have been identified as yet that would allow us to distinguish “functional” from “non-functional” binding events. With such major gaps in our understanding, it is perhaps no surprise that the study of TF function following on from ChIP-Seq map has proved difficult.

One major argument in favour of opportunistic binding has been the observation that cellular environments have a major impact on TF binding profiles, as indicated by the recurrent observation that many TFs show largely non-overlapping binding maps when assayed in two or more distinct cell types (Wei *et al*, 2011). Here, we have shown comprehensively that for nine TFs analysed in progenitor cells and mast cells, this was again the case. Moreover, a given factor was more similar to other factors in the same cell type rather than itself in a different cell type. Unlike previous studies however, we provide several lines of evidence to suggest that the different binding patterns in the two cell types are not largely the consequence of opportunistic non-functional binding because (i) differential binding can be used to construct a highly predictive model for “explaining” differential gene expression, (ii) consensus sequence motifs were overrepresented in both common and cell type specifically bound regions for all factors, (iii) knock-down of “shared” TFs in mast cells affected the expression of TF-bound and mast cell-specific expressed genes and (iv) mutation in consensus motifs for shared factors (ETS and GATA) reduced promoter activity in mast cell lines.

It is well recognised that TFs only bind a minor proportion of all possible matches to their consensus motif found in the genome (Bresnick *et al*, 2012). Moreover, factor binding is also commonly seen in regions that lack cognate binding sites. So-called master regulators have been shown to open new regulatory regions through binding to their consensus sequence motifs (Lichtinger *et al*, 2012; Mullen *et al*, 2011). Lineage regulators have also been implicated in mediating the co-localisation of signal-responsive TFs. For example, SMAD and TCF proteins, the downstream effector TFs of BMP and WNT signalling, were recently shown to be directed to cell type-specific enhancer regions by lineage-specific master regulators (Trompouki *et al*, 2011). Since interactions with specific DNA sequences appeared to play no major role in SMAD and TCF binding, the authors concluded that a major function of BMP/WNT signalling may be to reinforce the transcriptional state of a given cell, mediated largely through interactions of SMAD and TCF proteins with lineage-specific regulators. A recent study of the muscle determining TF MyoD argued against opportunistic binding as a general feature of key regulatory TFs (Yao *et al*, 2013). We believe that our experimental and computational analysis of 10 TFs in two related cell types provides an important new contribution to this ongoing debate. Moreover, our study constitutes the first comprehensive report of genome-wide TF binding profiles in mast cells and is therefore likely to have a major impact in furthering our understanding of the critical importance of mast cells for the functioning of the immune system.

## Materials and Methods

### Mouse bone marrow-derived mast cells (BMMCs)

Bone marrow cells were collected from tibias and femurs of 3- to 5-month-old mice, cultured in Iscove's modified Dulbecco's medium with 10% foetal bovine serum (Sigma), 1% penicillin/streptomycin (Sigma), 150 micromolar MTG (Sigma), 10% stem cell factor conditional media from BHK/MKL cells and 10 ng/ml of recombinant mIL-3 (Peprotech). Cells were frequently transferred to new flasks to remove adherent cells and experiments were performed after 3 weeks, when cultures were homogenous, as confirmed by the presence of FcERI by FACS and toluidine blue staining of cytospins (Supplementary Fig S12).

### Chromatin immunoprecipitation sequencing

ChIP assays were performed as previously described (Forsberg *et al*, 2000) using polyclonal antibodies against CTCF (Upstate, 07-729), E2A (Santa Cruz, sc-763x), ERG (Santa Cruz, sc354x), FLI1 (Abcam, ab15289-500), LMO2 (R&D, AF2726), MEIS1 (Santa Cruz, sc-10599x), PU.1 (Santa Cruz, sc-352x), RUNX1 (Abcam, ab23980-100), SCL/TAL1 (Santa Cruz, sc12984x), MITF (Cosmo Bio Co., BAM-73-107-EX), c-FOS (Santa Cruz, sc-253x), H3AcK27 (Abcam, ab4729) and control non-specific rabbit (Sigma, I5006) and goat (Sigma, I5256) IgG. ChIP samples were amplified and sequenced as described (Wilson *et al*, 2010). Reads were mapped to the mm9 mouse reference genome using Bowtie (Langmead *et al*, 2009) and peaks called using MACS (Zhang *et al*, 2008). Mapped reads were converted to density plots and displayed as UCSC genome browser custom tracks.

ChIP-Seq peak coordinates from all 10 shared TFs were combined into a single list and peaks overlapping by at least 1 bp were merged. A matrix was generated for peak coverage scores, denoted as cov(HPC-7) and cov(mast), for all the merged coordinates. Coverage scores were counted using the *intersectBed* function from BEDTools (Quinlan & Hall, 2010) and then normalised per 10 million reads. For *n* coordinates and 20 samples, this produces an *n *×* *20 coverage matrix. Hierarchical clustering and heatmap of the Pearson correlation coefficient between each pair of datasets were generated in *R*. This matrix was used for further analysis—differential score calculation and regression modelling.

### RNA sequencing

Total RNA was isolated using Tri-Reagent (Sigma-Aldrich) and digested with DNase I using TurboDNA (Applied Biosystems/Ambion). DNaseI-digested total RNA was processed and sequenced by BGI-Hong Kong. RNA-seq data were aligned to the mm9 transcriptome using TopHat (Trapnell *et al*, 2009) and assembled using Cufflinks (Trapnell *et al*, 2010). Expression values were expressed as *fpkm* (fragments per kilobase of exon per million fragments) values. Differential expression was calculated using Cuffdiff (Trapnell *et al*, 2010), and transcripts with *q*-value ≤ 0.05 were considered significant. Significant transcripts with *fpkm* values ≥ 2 in HPC7 and mast were classified as non-cell-type-specific (category I), while significant transcripts with absolute fold change ≥ 4 were classified as cell type-specific (categories II and III). Transcripts with *fpkm* values ≤ 2 were considered not expressed (category IV). Mapped reads were converted to density plots and displayed as UCSC genome browser custom tracks.

### Cell lines, reporter constructs, transfections and retroviral transduction

Murine mast cell lines FMP6- and MST have been described (McKinlay *et al*, 1998) (Elefanty & Cory, 1992) and characterised (Bockamp *et al*, 1998) previously. *Cx3cr1* luciferase reporter constructs were custom-made (Life Technologies), cloned into pGL2basic (Promega) and confirmed by sequencing. Cells were transfected and assayed as previously described (Gottgens *et al*, 1997).

shRNA fragments were inserted into pMSCV/LTRmiR30-PIG: luciferase (5′ CACGTACGCGGAATACTTCGAA 3′ (Bot *et al*, 2005)), Erg (5′ ACCTCCCAATATGACCACAAAT 3′), Gata2 (5′ CGCCGCCATTACTGTGAATATT 3′ (Huang *et al*, 2009)), Fli1 (5′ ACCAGTGAGAGTCAATGTCAAG 3′), Pu.1 (5′ AGGATGTGCTTCCCTTATCAAA 3′) and Lmo2 (5′ CCCAGCCCTTAGAGAGAATTTA 3′). Retrovirus was produced using the pCL-Eco Retrovirus Packaging Vector (Imgenex). BMMCs were infected with retrovirus by centrifugation at 2,200 rpm at 32°C for 1.5 h with 4 μg/ml polybrene (Sigma-Aldrich) after which the retroviral supernatant was replaced with fresh media. After 48 h, GFP^+^-transduced cells were sorted and cultured further for 24 h before RNA extraction. Knock-down efficiency is shown in Supplementary Fig S8A.

### Microarray gene expression analysis

DNaseI-digested RNA was processed and hybridised by Cambridge Genomic Services (Cambridge, UK). Samples were amplified using TotalPrep 96-RNA amplification kit (Applied Biosystems/Ambion) and hybridised to MouseWG-6 v2 microarrays (Illumina). Raw data were processed in *R* using variance stabilising transformation from the *lumi* package (Du *et al*, 2008). Probes with detection call ≥ 0.01 in all samples were removed, and then, the data were quantile-normalised. Differentially expressed probes between shRNA and control samples were identified using the *limma* package (Smyth, 2005). Probes with adjusted *P*-value ≤ 0.05 and fold change > 1.3 were considered significant.

### Gene set enrichment analysis

The BioGPS dataset was downloaded from NCBI Gene Expression Omnibus (GSE10246). CEL files were processed by *gcrma* algorithm, probes with “absent” call in all samples were removed, and remaining data were quantile-normalised in *R* using the *affy* package (Gautier *et al*, 2004). Gene set enrichment analysis (GSEA) (Subramanian *et al*, 2005) was performed on this expression dataset using only the following samples: CMP, mast cells, HSC, GMP, B cells, T cells, macrophages. Custom gene sets were generated from category II and III genes as described in the “RNA Sequencing” section.

### Statistical modelling

Differential scores were calculated for each of the 10 shared TFs and the RNA-seq data (equations 2012 and 1998). Differential gene expression (ΔGE) was calculated for each known gene transcript *t,* while differential TF scores (ΔTF) for a given TF were calculated for each peak region *r* in the coverage score matrix and then averaged for each gene. Peaks were assigned to genes using annotation from UCSC where peaks within ±1 kb of the transcription start site or within intragenic regions were assigned to the respective gene. The remaining intergenic peaks were assigned to both the nearest 5′ and 3′ genes. If no gene was found within 50 kb of an intergenic peak, it was not assigned to any genes. There were 151,925 TF-bound regions in total. 110,667 regions mapped to 19,163 genes and 41,258 regions did not map to any genes. Of 110,667 regions mapped to genes, 46,536 regions were bound by 2 or more TFs.



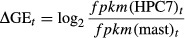
1

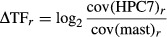
2

Linear regression models are useful for quantifying the relationships between multiple predictor variables to a response variable. In this study, regression models were employed to understand the relationship between quantitative changes in TF binding and changes in gene expression. Multiple linear regression models were analysed using the *stats* package in *R*. ΔTF_*i*_ are 10 predictor variables in the model corresponding to the 10 TFs, while ΔGE is the response variable for all genes *g* in the genome (equation 2005). Tenfold cross-validation was carried out to obtain Pearson's correlation coefficient between predicted and observed values. 10% of the entire dataset was used for testing, while 90% was used for training. The procedure was repeated until all the data have been utilised for testing and each repetition has non-overlapping test dataset. *R*^2^ is the squared correlation coefficient and prediction accuracy was taken as the average of all *R*^2^ values. Scatterplot correlation coefficients were calculated from the observed and predicted values from fitting the full dataset.

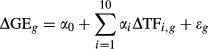
3

Another modelling approach used in this study is the generalised additive models (GAMs). This type of model uses the sum of smooth functions ∑s(X_*n*_) instead of the linear forms ∑β_*n*_X_*n*_ to allow non-linear relationships between the predictor variables (X_1_, X_2_, X_3_,…, X_*n*_) and the response variable. GAMs with and without interaction terms were analysed using the *mgcv* package in *R* (Wood, 2011). *mgcv* uses smoothing spline functions of the predictor variables. Equation 2008 corresponds to the GAM with interaction term where we included all pairwise interaction terms (ΔTF_*i*_*,* ΔTF_*j*_) between the 10 factors. Only genes with at least 2 TFs bound were used for this analysis since genes bound by 1 TF are not suitable for testing interactions. Differential gene expression (ΔGE) is assumed to have a Gaussian distribution with an identity link function. Restricted maximum-likelihood (REML) (Wood, 2011) estimation was used in selecting the smoothing parameters, and adjusted *R*^2^ values was used to assess model prediction accuracy.

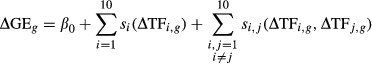
4

### Motif analysis

*De novo* motif analysis on the central 100-bp peak regions was carried out using the Homer software (Heinz *et al*, 2010), and matches to known motifs were discovered using TOMTOM (Gupta *et al*, 2007). Similarity to known motifs was discovered from 2 databases—Jaspar and UniProbe (Bryne *et al*, 2008; Robasky & Bulyk, 2011). Only significant motifs (*q*-value ≤ 0.01) were reported. To carry out the motif content analysis, the same procedure as above was carried out on cell type-specific and common peak regions. Peak regions for each of the 10 shared TFs were divided into three categories: HPC7-specific (HPC7-bound only), mast cell-specific (mast-bound only) and common (HPC7/mast peaks overlapping ≥ 1 bp) regions. Results from *de novo* motif analysis were combined in a matrix where columns are datasets, rows are Jaspar/UniProbe motifs and each row/column value is *x* or 0, where *x* corresponds to the fraction of peaks with significant (TOMTOM *q*-value ≤ 0.05) match to a particular motif, while 0 is a non-significant match. The matrix was used to generate a heatmap that indicates fraction of peaks containing motif in cell type-specific and common regions.

### Plotting in *R*

Bar charts and scatterplots were plotted using *ggplot2* (Wickham, 2009). Venn diagrams were created using the *venneuler* package (Wilkinson, 2011). Heatmaps were generated using the *gplots* packages (Warnes *et al*, 2012).

### Data deposition

The raw and processed data from the ChIP-Seq, RNA-Seq and microarray experiments reported in this publication have been submitted to the NCBI Gene Expression Omnibus (http://www.ncbi.nlm.nih.gov/geo) and assigned the identifier GSE48086.
