# Supplementary material for: Key regulators control distinct transcriptional programmes in blood progenitor and mast cells
Source: EMBO J. 2014 Apr 23;33(11):1212–26. doi: 10.1002/embj.201386825 (PMC4168288; doi:10.1002/embj.201386825)
Supplement: Supplementary file 1 [file embj0033-1212-sd1.pdf]

Figure S1

A

Sample	Replicate	# of uniquely mappable reads
HPC7	1	31,729,039
HPC7	2	32,241,516
Mast	1	31,712,993
Mast	2	34,089,097

B

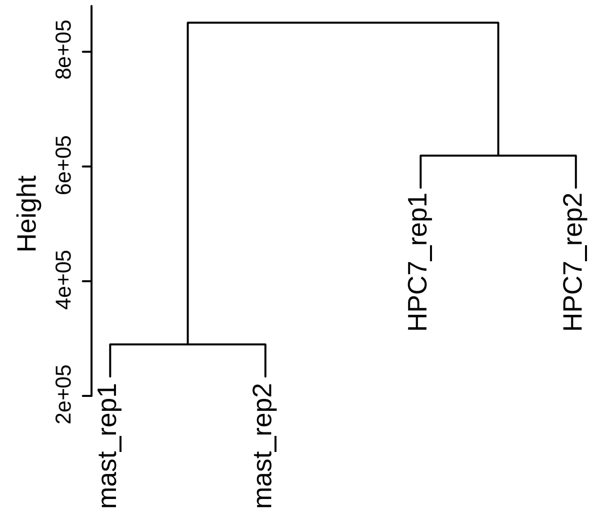

C

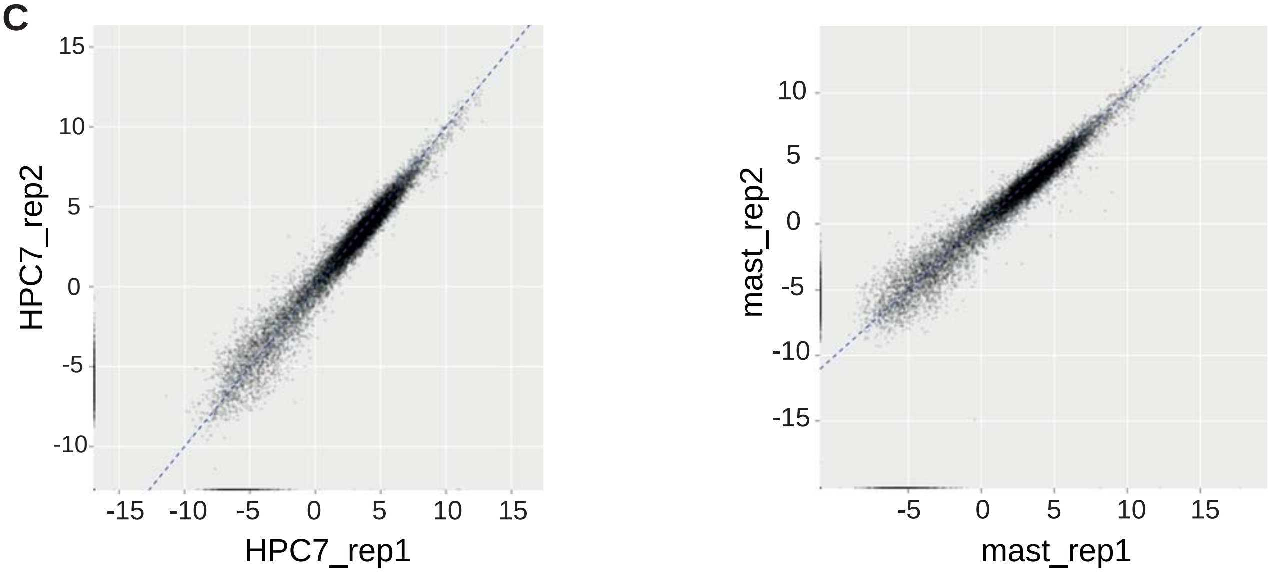

Figure S1 – RNA-seq data quality control. (A) Number of uniquely mappable reads for all replicates and samples. (B) Hierarchical clustering of the RNA-seq datasets. (C) Scatterplot between replicate datasets.
