# Supplementary material for: Key regulators control distinct transcriptional programmes in blood progenitor and mast cells
Source: EMBO J. 2014 Apr 23;33(11):1212–26. doi: 10.1002/embj.201386825 (PMC4168288; doi:10.1002/embj.201386825)
Supplement: Supplementary file 2 [file embj0033-1212-sd2.pdf]

Figure S2

A

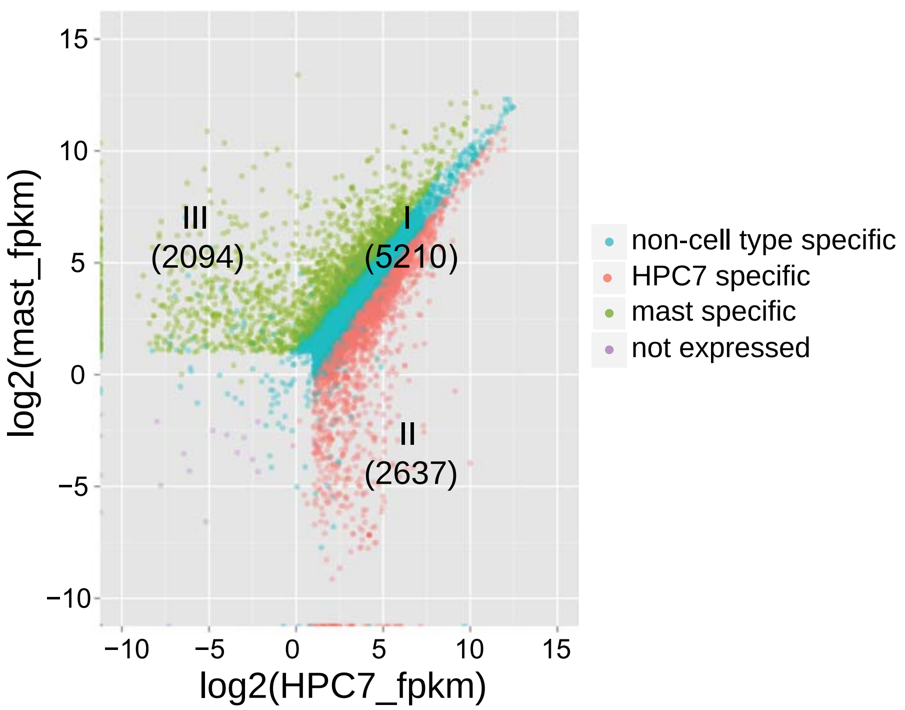
15
10
5
0
−5
−10
−10
−5
0
5
10
15
III
(2094)
I
(5210)
II
(2637)
log2(mast_fpkm)
log2(HPC7_fpkm)
non-cell type specific
HPC7 specific
mast specific
not expressed

B

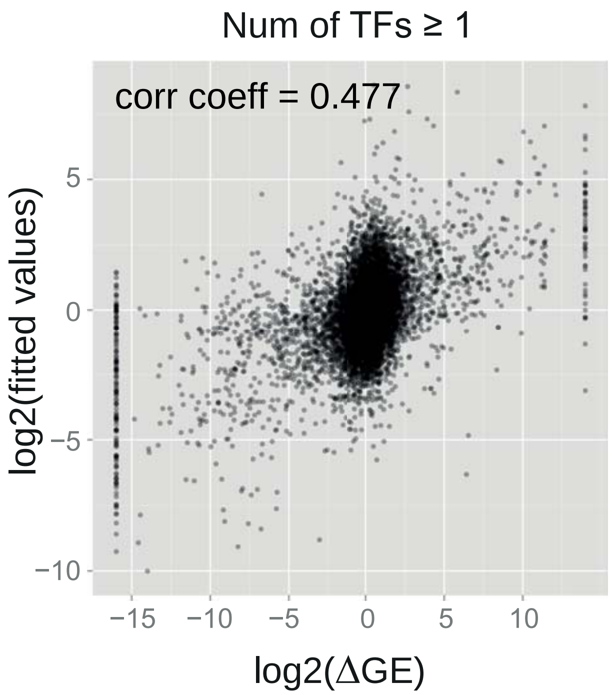
Num of TFs ≥ 1
corr coeff = 0.477
5
0
−5
−10
−15
−10
−5
0
5
10
log2(fitted values)
log2(ΔGE)

Figure S2 – Mathematical modelling of gene expression and transcription factor variability. (A) Scatterplot representing expression (in fpkm values obtained by RNA-seq) for genes bound by at least 1 TF in HPC7 and mast cells used for mathematical modelling. Each dot on the scatterplot is coloured based on 4 categories: (I) non-cell-type specific, (II) HPC7-specific, (III) mast-specific and (IV) not expressed. 58.6% of genes expressed in either cell type are bound by at least 1 shared TF. These genes show similar distribution of expression scores as the entire transcriptome shown in Figure 1. (B) Scatterplot of observed and predicted values from the multiple linear regression model for genes bound by at least 1 shared TF. The range of predicted values for genes with no observed change in expression is very large (predicted ΔGE range from -5 to 5 for ΔGE~0).
