# Supplementary figures and images for: Key regulators control distinct transcriptional programmes in blood progenitor and mast cells
Source: EMBO J. 2014 Apr 23;33(11):1212–26. doi: 10.1002/embj.201386825 (PMC4168288; doi:10.1002/embj.201386825)

**Figure S3**

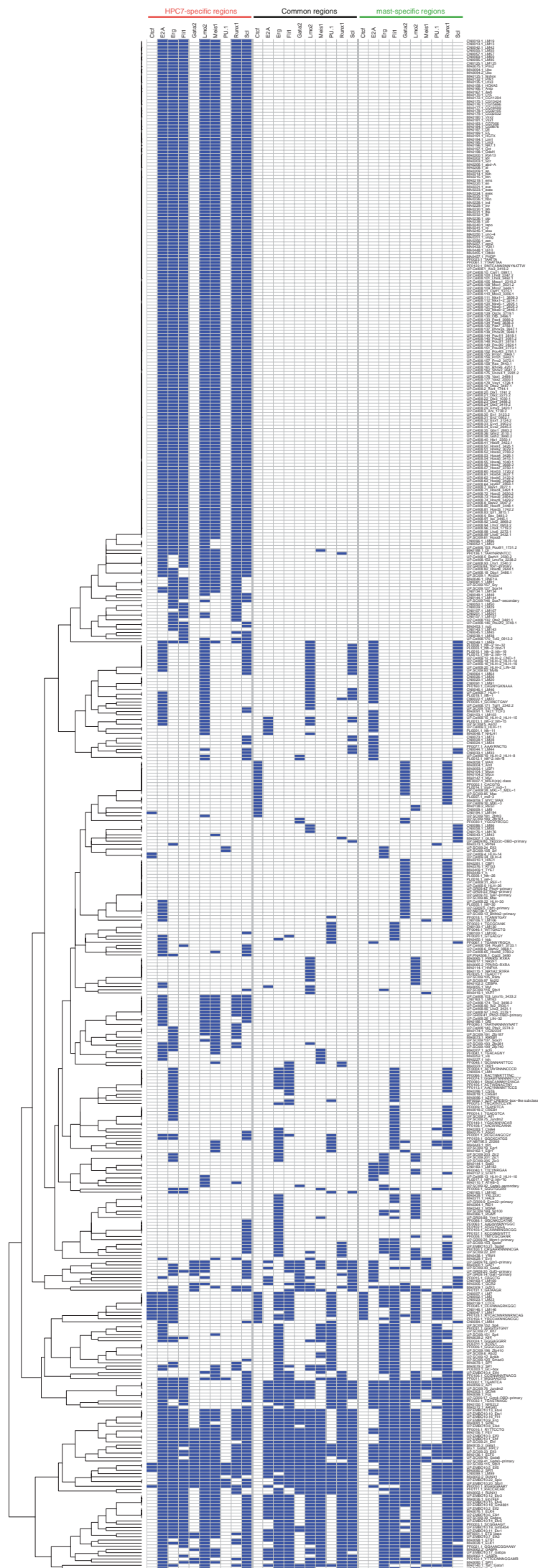

**Figure S3** – Full results heatmap from the motif content analysis.

Supplement: Supplementary file 3 [file embj0033-1212-sd3.pdf]
