# Supplementary material for: Key regulators control distinct transcriptional programmes in blood progenitor and mast cells
Source: EMBO J. 2014 Apr 23;33(11):1212–26. doi: 10.1002/embj.201386825 (PMC4168288; doi:10.1002/embj.201386825)
Supplement: Supplementary file 4 [file embj0033-1212-sd4.pdf]

Figure S4

Generalized Additive Model (12TFs)

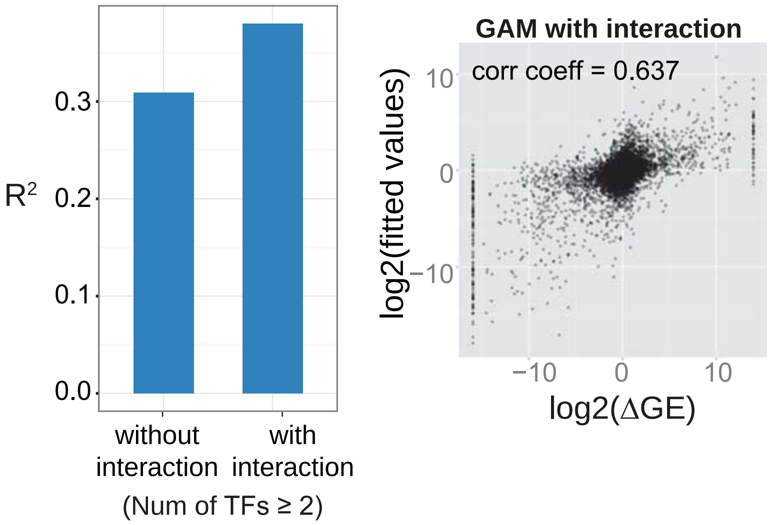

Figure S4 – Generalized Additive Model (GAM) including Mitf and c-Fos. Average R^2 values with standard error for GAM on all genes bound by ≥ 2 transcription factors based on 10-fold cross validation. The scatterplot shows the correlation between observed and predicted values in this model. Since Mitf is not expressed in HPC7 cells and c-Fos has low expression, we utilized an IgG ChIP-seq to represent background levels of these 2 TFs in HPC7 so that differential binding can be quantified between mast and HPC7 cells. Although our GAM model including 12 TFs did not show an increase in R^2 values, it incorporates ~700 more genes (see results in Supplemenrary Tables S8 and S9).
