# Supplementary material for: Key regulators control distinct transcriptional programmes in blood progenitor and mast cells
Source: EMBO J. 2014 Apr 23;33(11):1212–26. doi: 10.1002/embj.201386825 (PMC4168288; doi:10.1002/embj.201386825)
Supplement: Supplementary file 5 [file embj0033-1212-sd5.pdf]

Figure S5

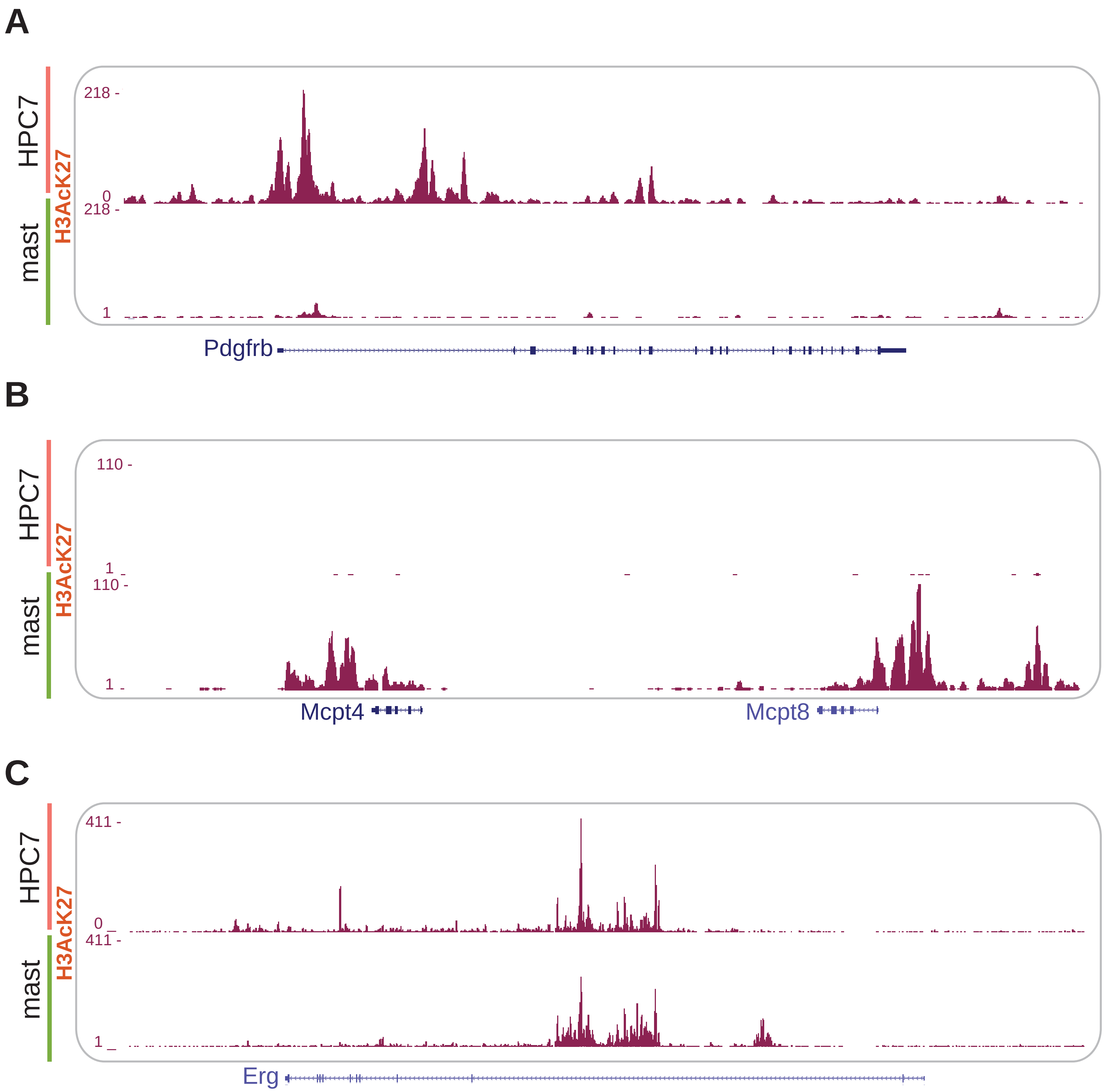

Figure S5 – Acetylation of Lysine 27 of histone H3 (H3AcK27) profile in mast and HPC-7 cells. Snap shots corresponding to active regions in HPC-7 (A), mast (B) or both cell types (C) are shown.
