# Supplementary material for: Key regulators control distinct transcriptional programmes in blood progenitor and mast cells
Source: EMBO J. 2014 Apr 23;33(11):1212–26. doi: 10.1002/embj.201386825 (PMC4168288; doi:10.1002/embj.201386825)
Supplement: Supplementary file 6 [file embj0033-1212-sd6.pdf]

Figure S6

A

All genes

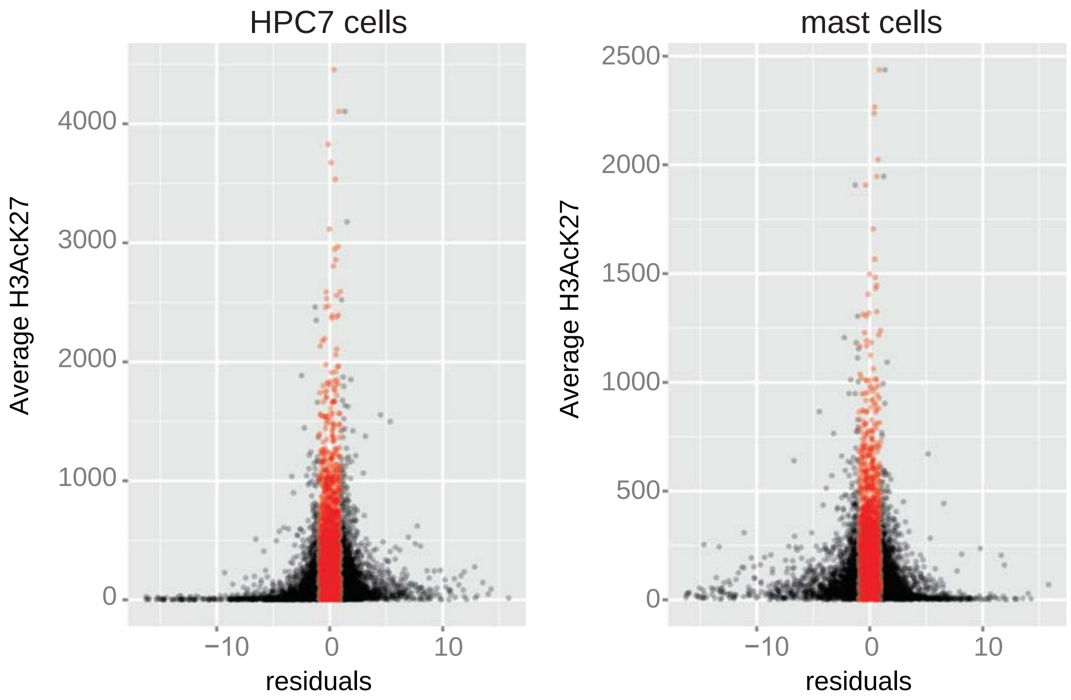

	Low H3AcK27	High H3AcK27
● **residual > 1.5**	1512	849
● **residual ≤ 1.5**	2600	3269

Chi-square test p-value < 2.2e-16

B

Differentially expressed genes

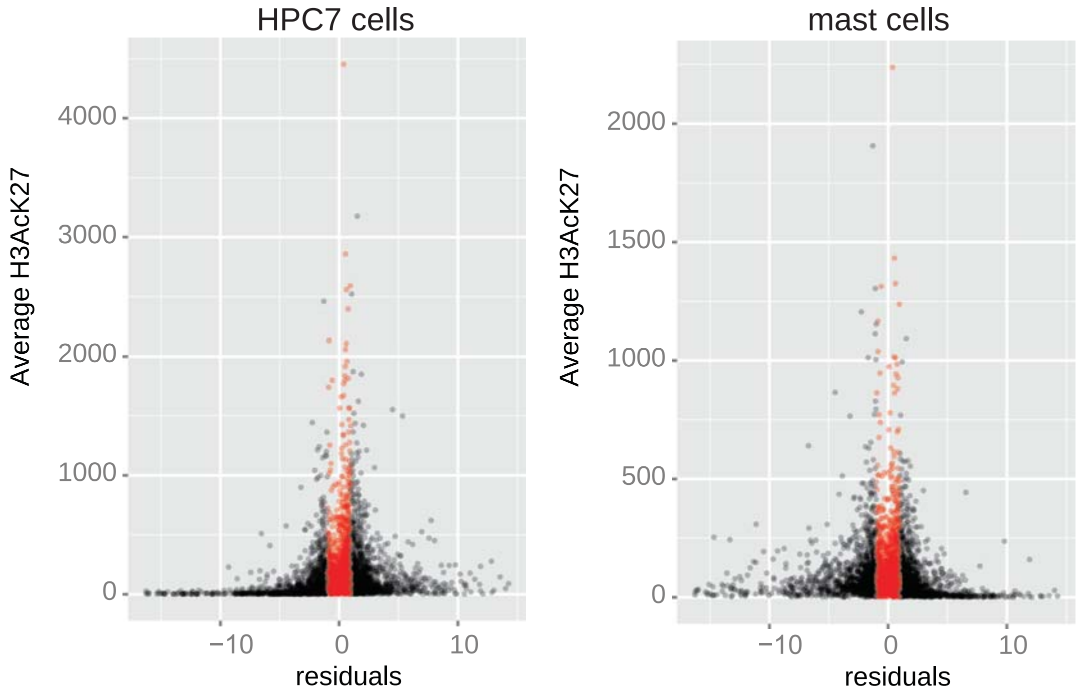

	Low H3AcK27	High H3AcK27
● **residual > 1.5**	1167	612
● **residual ≤ 1.5**	1008	1116

Chi-square test p-value < 2.2e-16

Figure S6 – Relationship between genes predicted well by the GAM (≥2TFs) and H3AcK27. Scatterplots comparing average H3AcK27 reads with GAM prediction (residuals) for all genes used for modelling (A) and only those genes that are differentially expressed either in HPC7 o mast cells (B) are shown. The numbers of H3AcK27 reads for any given gene were averaged across all TF bound regions (peaks) assigned to that gene (see 'Materials and methods' for peak to gene assignment). Data points are coloured red for genes with absolute residuals ≤1.5 and black otherwise. Genes were considered to have 'high H3AcK27' if the average number of reads was ≥128 reads in at least one cell type. Chi-square test results are also shown.
