# Supplementary material for: Key regulators control distinct transcriptional programmes in blood progenitor and mast cells
Source: EMBO J. 2014 Apr 23;33(11):1212–26. doi: 10.1002/embj.201386825 (PMC4168288; doi:10.1002/embj.201386825)
Supplement: Supplementary file 7 [file embj0033-1212-sd7.pdf]

Figure S7

A

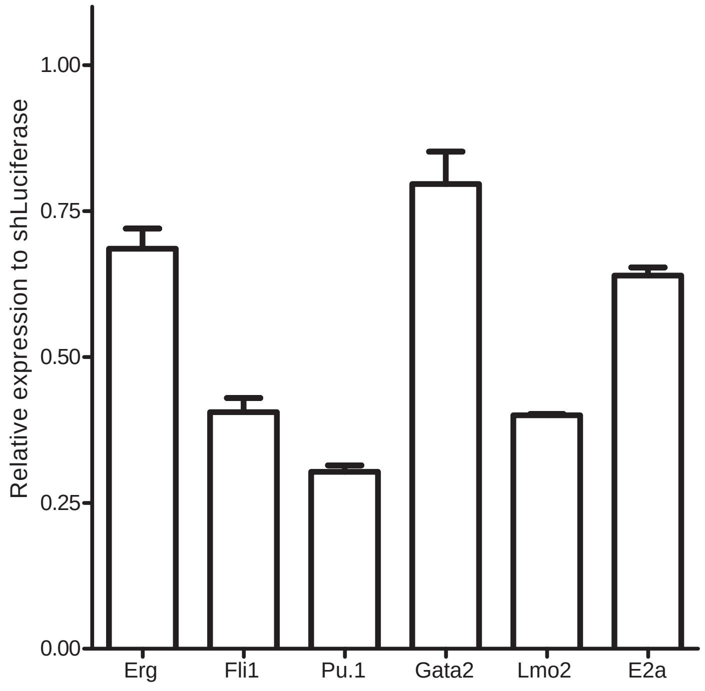
Relative expression to shLuciferase
1.00
0.75
0.50
0.25
0.00
Erg
Fli1
Pu.1
Gata2
Lmo2
E2a

B

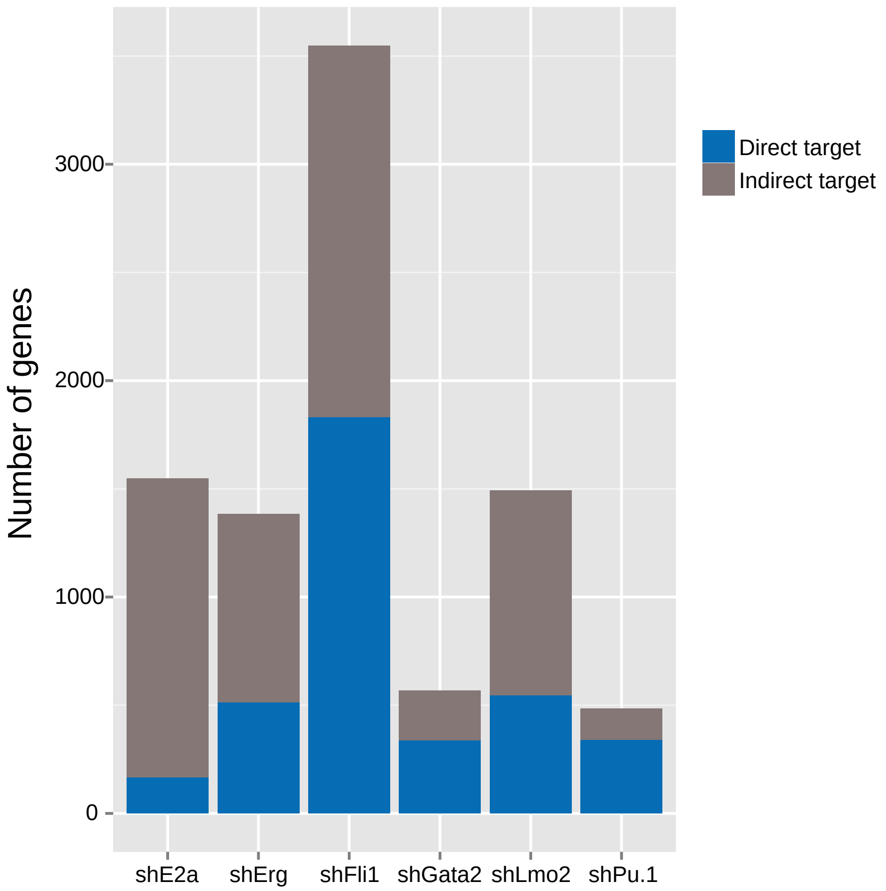
Number of genes
3000
2000
1000
0
shE2a
shErg
shFli1
shGata2
shLmo2
shPu.1
Direct target
Indirect target

Figure S7 – Knock-down experiments in primary mast cells. (A) Knock-down efficiency over multiple independent biological replicate experiments. Expression levels of Erg, Fli1, Pu.1, Gata2, Lmo2 and E2a in primary mast cells was measured by qPCR following knock-down of the corresponding factor. (B) Differentially expressed genes (p-value < 0.05, fold change > 1.3) following TF knock-down that contained binding peaks in their loci for the respective TF. For each TF, the bar chart indicates the total number of genes that changed expression following knock-down of the indicated factor and the fraction of genes that were directly bound by the indicated factor (direct target).
