# Supplementary material for: Key regulators control distinct transcriptional programmes in blood progenitor and mast cells
Source: EMBO J. 2014 Apr 23;33(11):1212–26. doi: 10.1002/embj.201386825 (PMC4168288; doi:10.1002/embj.201386825)
Supplement: Supplementary file 8 [file embj0033-1212-sd8.pdf]

Figure S8

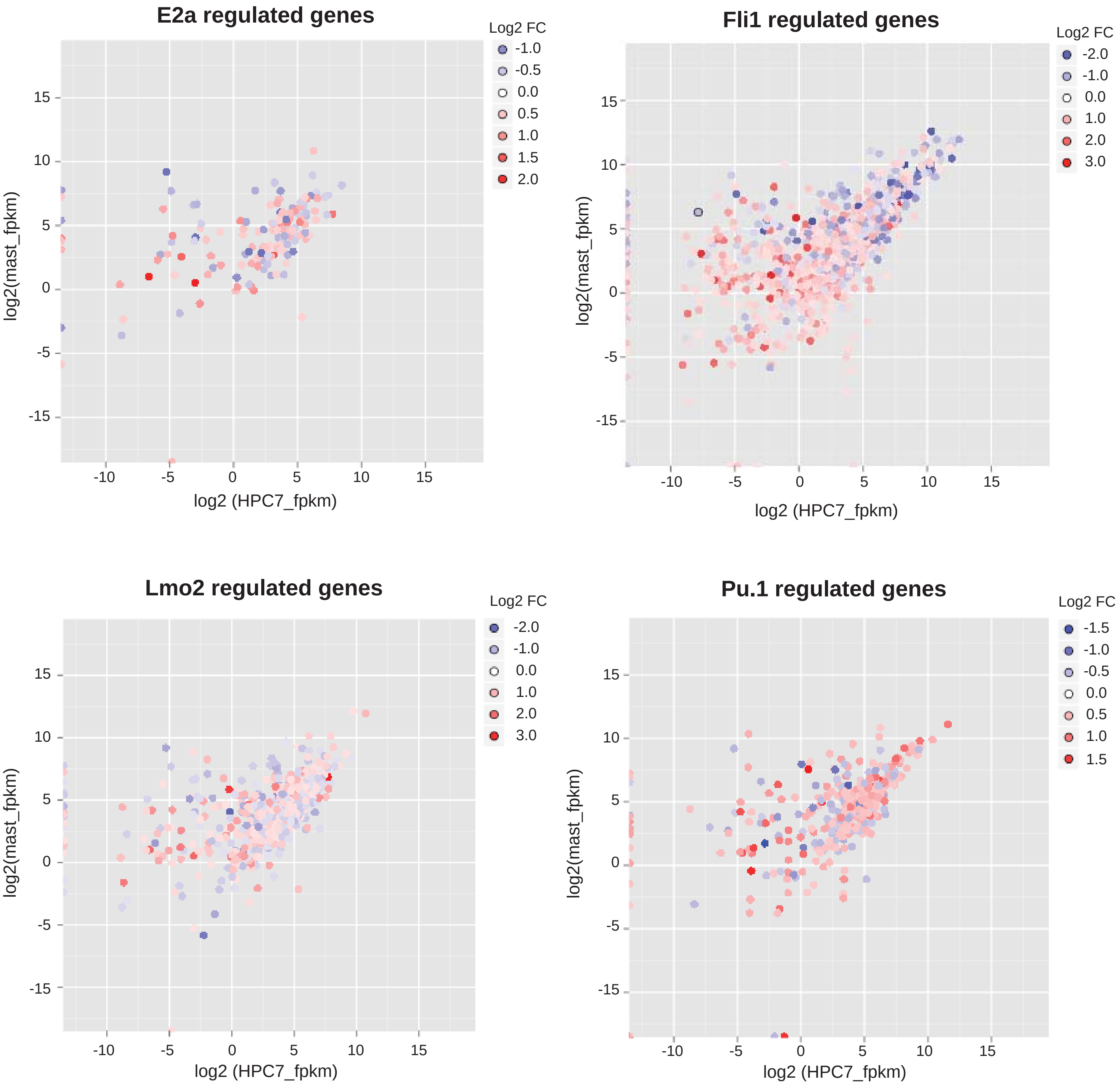

Figure S8 – Perturbation of key haematopoietic TFs, E2a, Fli1, Lmo2 and Pu.1. Scatterplots show E2a, Fli1, Lmo2 and Pu.1 regulated targets. Points on the scatter plot are coloured based on the log2 fold change of shRNA compared to control. Only genes that are differentially expressed in the knock down (absolute log FC>0.38, p-value≤0.05) are shown. The complete list of regulated targets and expression values can be found in Table S10.
