# Supplementary material for: Key regulators control distinct transcriptional programmes in blood progenitor and mast cells
Source: EMBO J. 2014 Apr 23;33(11):1212–26. doi: 10.1002/embj.201386825 (PMC4168288; doi:10.1002/embj.201386825)
Supplement: Supplementary file 9 [file embj0033-1212-sd9.pdf]

# Figure S9

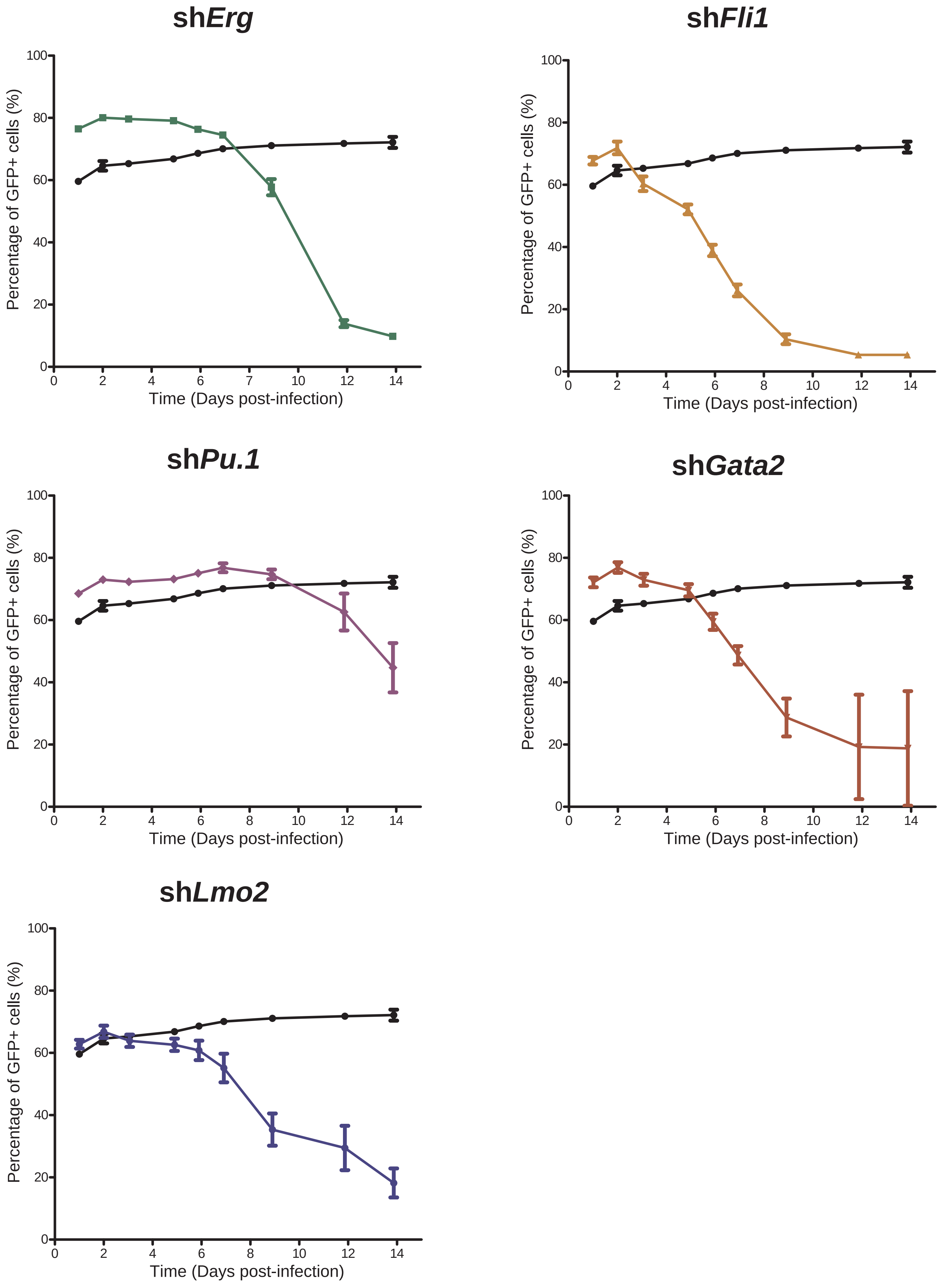

shErg
100
80
60
40
20
0
0
2
4
6
7
10
12
14
Time (Days post-infection)
Percentage of GFP+ cells (%)
shFli1
100
80
60
40
20
0
0
2
4
6
8
10
12
14
Time (Days post-infection)
Percentage of GFP+ cells (%)
shPu.1
100
80
60
40
20
0
0
2
4
6
8
10
12
14
Time (Days post-infection)
Percentage of GFP+ cells (%)
shGata2
100
80
60
40
20
0
0
2
4
6
8
10
12
14
Time (Days post-infection)
Percentage of GFP+ cells (%)
shLmo2
100
80
60
40
20
0
0
2
4
6
8
10
12
14
Time (Days post-infection)
Percentage of GFP+ cells (%)

**Figure S9** – Effect of knock-down of shared TFs in MST mast cell line growth. MST cells were transfected with constructs that contained shRNAs against Erg, Fli1, Gata2, Pu.1 or Lmo2 and expression cassette for GFP. Transfected cells expressed simultaneously shRNA and GFP. After infection, we maintained in culture infected and uninfected cells together and we monitored the percentage of GFP expressing cells by FACs for 2 weeks. Black lines correspond to the control (shLuciferase) while coloured lines correspond to the indicated TF. Reduction of levels of shared TFs had a pronounced growth effect when in competition with wild type cells. Representative experiment is shown. Standard error is depicted.
