# Supplementary material for: Key regulators control distinct transcriptional programmes in blood progenitor and mast cells
Source: EMBO J. 2014 Apr 23;33(11):1212–26. doi: 10.1002/embj.201386825 (PMC4168288; doi:10.1002/embj.201386825)
Supplement: Supplementary file 10 [file embj0033-1212-sd10.pdf]

Figure S10

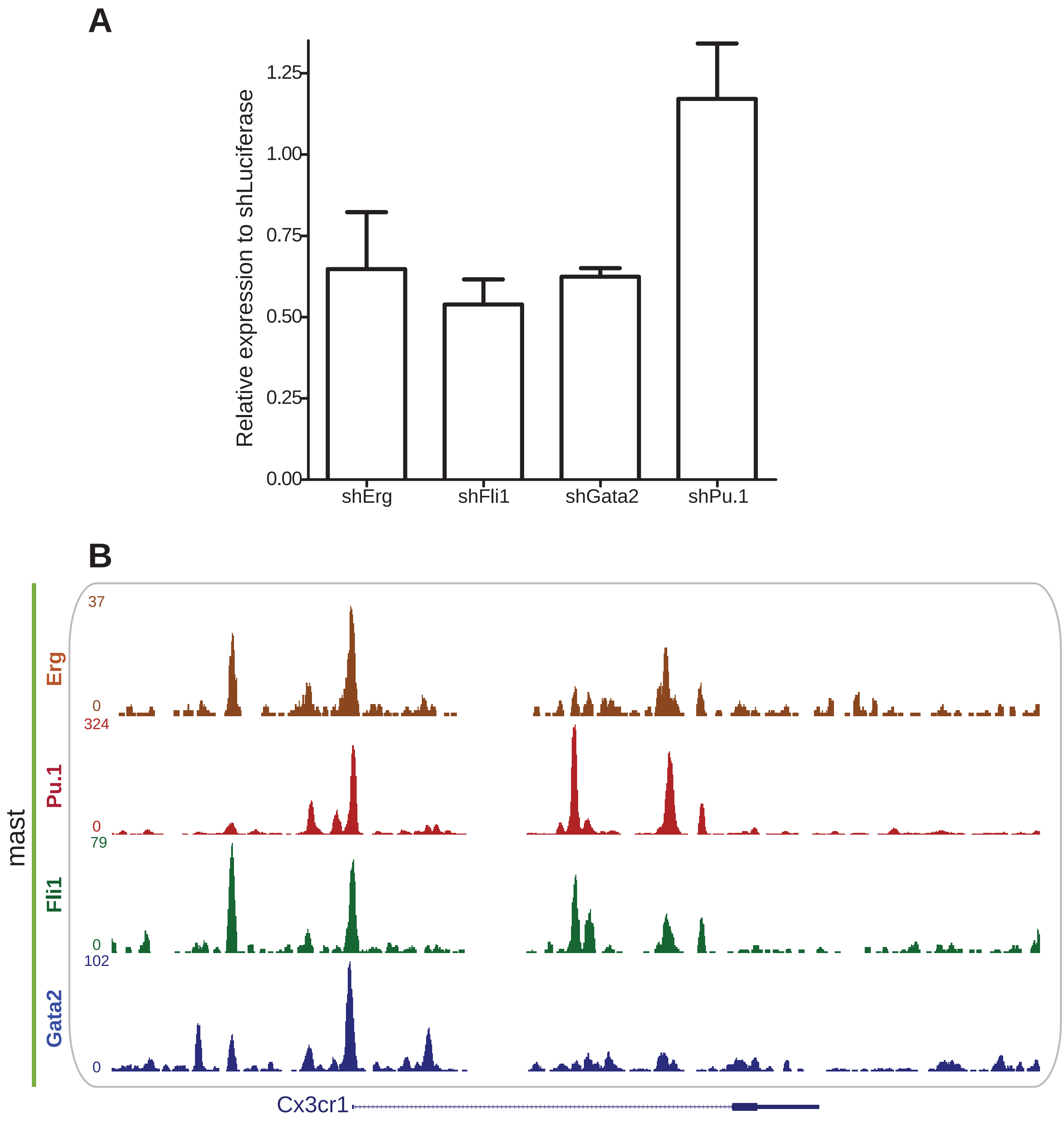

Figure S10 – Direct control of mast cell specific genes by shared TFs. (A) Expression levels of *Cx3cr1* following knock-down of Erg, Fli1, Pu.1 and Gata2 in primary mast cells. (B) Genome browser screenshot of the mast-cell-specific gene *Cx3cr1*. Binding of ERG, FLI1, PU.1 and GATA2 to the promoter region is shown.
