# Supplementary material for: Key regulators control distinct transcriptional programmes in blood progenitor and mast cells
Source: EMBO J. 2014 Apr 23;33(11):1212–26. doi: 10.1002/embj.201386825 (PMC4168288; doi:10.1002/embj.201386825)
Supplement: Supplementary file 11 [file embj0033-1212-sd11.pdf]

Figure S11

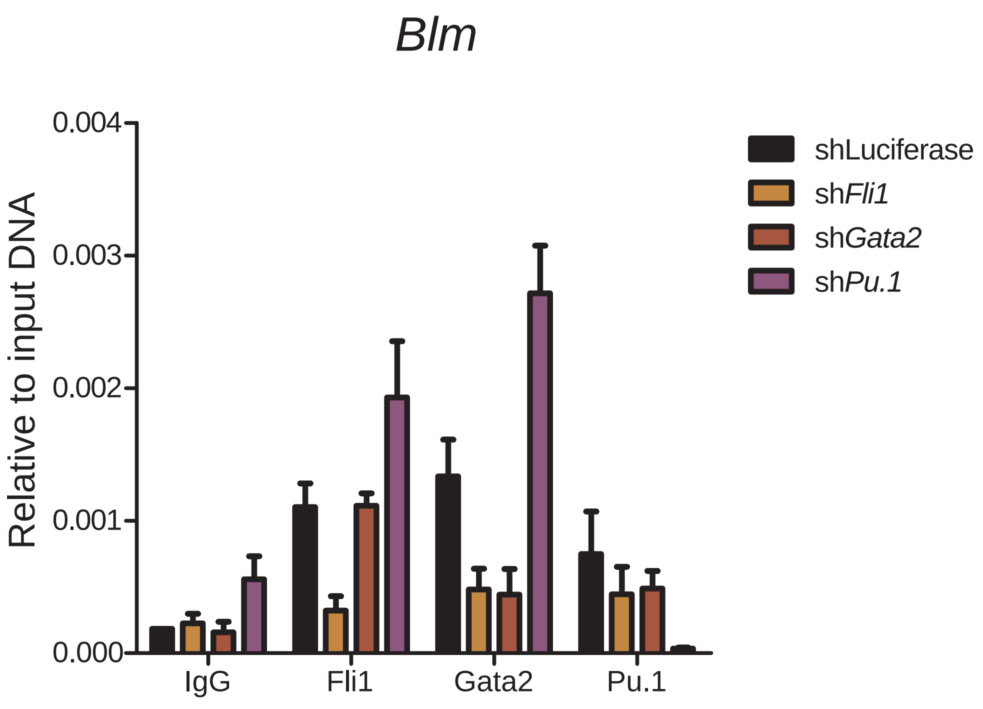
Blm
Relative to input DNA
0.004
0.003
0.002
0.001
0.000
IgG
Fli1
Gata2
Pu.1
shLuciferase
shFli1
shGata2
shPu.1

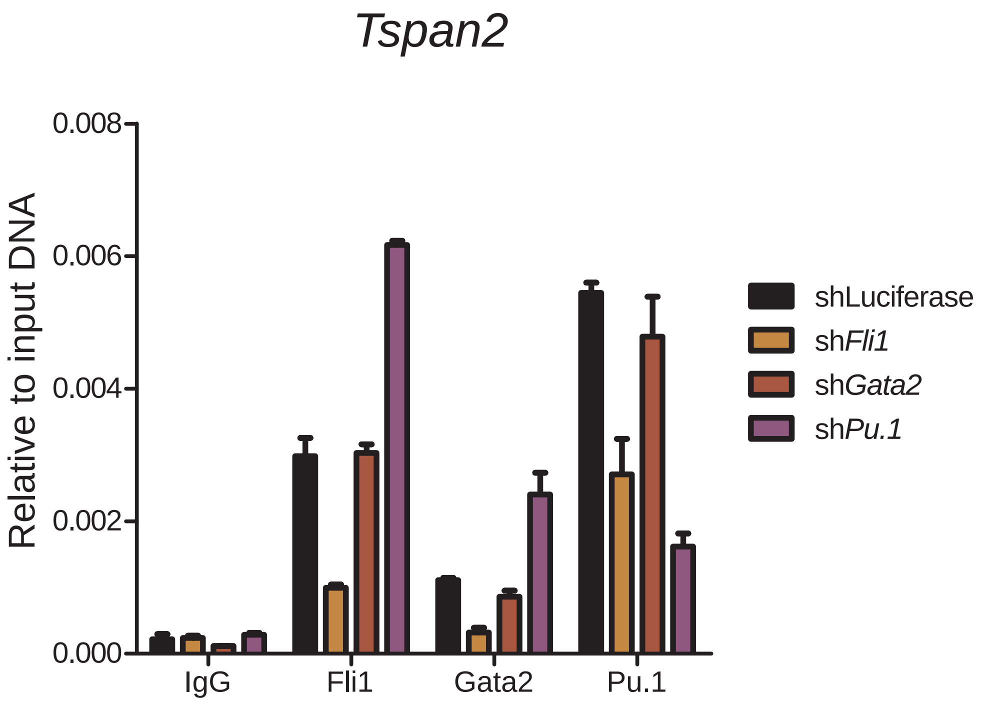
Tspan2
Relative to input DNA
0.008
0.006
0.004
0.002
0.000
IgG
Fli1
Gata2
Pu.1
shLuciferase
shFli1
shGata2
shPu.1

Figure S11 – Real-time PCR analysis of ChIP for FLI1, GATA2 and PU.1 following knock-down of the same genes in regions associated to the genes *Blm and Tspan2*. Experiments were performed in MST mast cell line and results are expressed relative to total DNA input. Primers used for amplifications were: Blm (5' GGGATGAGTCACTGCCAACT 3' and 5' ATTTGCCTTGTTTCCTGCTG 3'); Tspan2 (5' GCCCCATCTAGGTCTGTTCA 3' and 5' ACTCTGGCATTCAGGCACTT 3').
