# Supplementary material for: Key regulators control distinct transcriptional programmes in blood progenitor and mast cells
Source: EMBO J. 2014 Apr 23;33(11):1212–26. doi: 10.1002/embj.201386825 (PMC4168288; doi:10.1002/embj.201386825)
Supplement: Supplementary file 12 [file embj0033-1212-sd12.pdf]

Figure S12

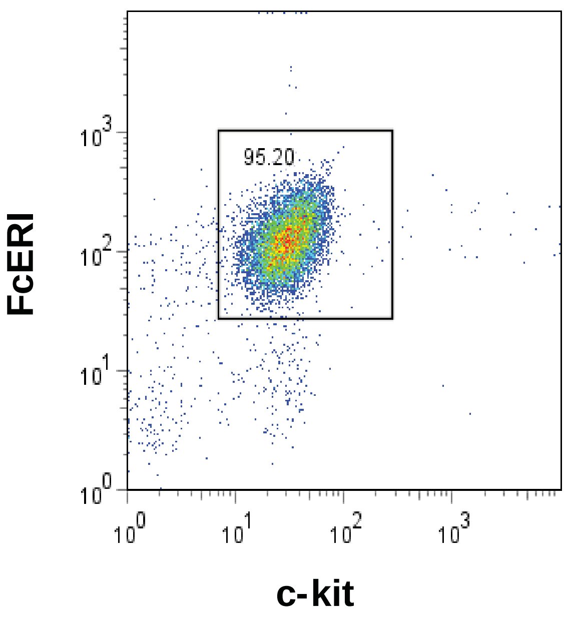

Figure S12 – Representative FACS plot for c-kit and FcERI expression in mouse bone marrow derived mast cells after 3 weeks of culture.
