# Supplementary material for: Key regulators control distinct transcriptional programmes in blood progenitor and mast cells
Source: EMBO J. 2014 Apr 23;33(11):1212–26. doi: 10.1002/embj.201386825 (PMC4168288; doi:10.1002/embj.201386825)
Supplement: Supplementary file 13 [file embj0033-1212-sd13.pdf]

Gene Set: RNAseq_HPC7_UP-REG

Up-reg in phenotype	NES	p-value
HSC	2.99	<0.001
CMP	2.97	<0.001
GMP	2.82	<0.001
B-cells	2.66	<0.001
T-cells	2.25	<0.001
Macrophage	2.25	<0.001

Table S1 – Comparison of HPC7 gene set enrichment in various haematopoietic cell types. Enrichment of HPC7 up-regulated genes was assessed by Gene Set Enrichment Analysis (GSEA) on expression data of haematopoietic progenitor as well as differentiated cell types from BioGPS. NES denotes normalized enrichment score.
