# Supplementary material for: Key regulators control distinct transcriptional programmes in blood progenitor and mast cells
Source: EMBO J. 2014 Apr 23;33(11):1212–26. doi: 10.1002/embj.201386825 (PMC4168288; doi:10.1002/embj.201386825)
Supplement: Supplementary file 14 [file embj0033-1212-sd14.pdf]

	# of peaks		
**Transcription factor**	**HPC7 only**	**mast only**	**HPC7 and mast**
CTCF	13438	11184	30508
E2A	21363	1447	1154
Erg	23687	4191	11433
Fli1	11465	17250	8017
Gata2	5350	24731	3770
Lmo2	6508	10458	3010
Meis1	7354	820	595
PU.1	9859	35249	11805
Runx1	2225	32074	2977
Scl	5573	3998	1510

Table S2 – Number of unique and overlapping ChIP-Seq peaks in HPC7 and mast. Peaks are shared between HPC7 and mast if they have at least 1bp overlap.
