# Supplementary material for: Key regulators control distinct transcriptional programmes in blood progenitor and mast cells
Source: EMBO J. 2014 Apr 23;33(11):1212–26. doi: 10.1002/embj.201386825 (PMC4168288; doi:10.1002/embj.201386825)
Supplement: Supplementary file 15 [file embj0033-1212-sd15.pdf]

Minimum # of TFs	# of genes tested	R^2 (cross val)	p-val	AIC
1	9952	0.226569095	< 2.2e-16	48455.43
2	8261	0.252272577	< 2.2e-16	40207.77
3	5840	0.290425152	< 2.2e-16	29027.05
4	3041	0.359266515	< 2.2e-16	15752.11
5	1223	0.414356927	< 2.2e-16	6821.309

Table S3 – Multiple Linear Regression Model results. '# of genes tested' indicates the number of genes that satisfy the criteria of minimum number of TFs bound per gene. 'R2 (cross val)' refers to the average R^2 values obtained from 10-fold cross validation and 'AIC' refers to the Akaike Information Criterion of the model.
