# Supplementary material for: Key regulators control distinct transcriptional programmes in blood progenitor and mast cells
Source: EMBO J. 2014 Apr 23;33(11):1212–26. doi: 10.1002/embj.201386825 (PMC4168288; doi:10.1002/embj.201386825)
Supplement: Supplementary file 16 [file embj0033-1212-sd16.pdf]

Significance codes: 0.001 → ‘***’, 0.01 → ‘**’, 0.05 → ‘*’, 0.1 → ‘.’, 1 → ‘’

Model	Predictor Variables	Estimate	Std. Error	t value	Pr(>|t|)	Significance
**MLR (≥1 TF)**	(Intercept)	0.26166	0.065	4.025	5.73e-05	***
	Ctcf	0.41388	0.02583	16.023	<2e-16	***
	E2a	0.22215	0.01966	11.301	<2e-16	***
	Erg	0.61305	0.02704	22.669	<2e-16	***
	Fli1	-0.01769	0.01466	-1.207	0.22753	
	Gata2	0.11963	0.01595	7.498	7.03e-14	***
	Lmo2	-0.01234	0.0126	-0.98	0.32731	
	Meis1	-0.0919	0.02795	-3.287	0.00101	**
	Pu.1	0.28685	0.01843	15.561	<2e-16	***
	Runx1	0.04541	0.01582	2.87	0.00411	**
	Scl	0.16626	0.03004	5.535	3.20e-08	***
**MLR (≥2 TF)**	(Intercept)	0.32925	0.0889	3.703	0.000214	***
	Ctcf	0.43205	0.02803	15.416	<2e-16	***
	E2a	0.20766	0.02105	9.866	<2e-16	***
	Erg	0.65385	0.02934	22.284	<2e-16	***
	Fli1	-0.02635	0.01625	-1.622	0.104886	
	Gata2	0.12273	0.01677	7.319	2.74e-13	***
	Lmo2	-0.01576	0.01284	-1.227	0.219706	
	Meis1	-0.10659	0.02958	-3.604	0.000315	***
	Pu.1	0.29265	0.02001	14.624	<2e-16	***
	Runx1	0.05176	0.0186	2.783	0.005393	**
	Scl	0.16775	0.03087	5.434	5.66e-08	***
**MLR (≥3 TF)**	(Intercept)	0.20107	0.12001	1.675	0.093909	.
	Ctcf	0.43624	0.03342	13.054	<2e-16	***
	E2a	0.2132	0.02533	8.417	<2e-16	***
	Erg	0.77302	0.03545	21.805	<2e-16	***
	Fli1	-0.05223	0.02066	-2.529	0.011471	*
	Gata2	0.07115	0.02041	3.486	0.000494	***
	Lmo2	-0.04187	0.01509	-2.774	0.005553	**
	Meis1	-0.09002	0.03474	-2.591	0.009584	**
	Pu.1	0.31641	0.02397	13.2	<2e-16	***
	Runx1	0.07191	0.02429	2.961	0.00308	**
	Scl	0.19272	0.03493	5.517	3.59e-08	***
**MLR (≥4 TF)**	(Intercept)	-0.26991	0.18697	-1.444	0.14896	
	Ctcf	0.46111	0.04709	9.793	<2e-16	***
	E2a	0.28081	0.03605	7.788	9.26e-15	***
	Erg	0.94411	0.05014	18.828	<2e-16	***
	Fli1	-0.06052	0.03253	-1.861	0.06291	.
	Gata2	-0.02391	0.03296	-0.725	0.46821	
	Lmo2	-0.09261	0.02358	-3.927	8.80e-05	***
	Meis1	-0.07741	0.04913	-1.576	0.11518	
	Pu.1	0.31188	0.0353	8.834	<2e-16	***
	Runx1	0.1124	0.03775	2.978	0.00293	**
	Scl	0.18811	0.04449	4.228	2.43e-05	***
**MLR (≥5 TF)**	(Intercept)	-0.96822	0.34352	-2.818	0.004904	**
	Ctcf	0.53405	0.08665	6.163	9.68e-10	***
	E2a	0.34539	0.06023	5.734	1.24e-08	***
	Erg	1.06742	0.08513	12.538	<2e-16	***

	Fli1	-0.03742	0.06156	-0.608	0.543388	
	Gata2	-0.13627	0.07387	-1.845	0.065324	.
	Lmo2	-0.14619	0.04848	-3.016	0.002618	**
	Meis1	-0.01981	0.08123	-0.244	0.807376	
	Pu.1	0.25189	0.06467	3.895	0.000103	***
	Runx1	0.17698	0.07179	2.465	0.013836	*
	Scl	0.15405	0.06996	2.202	0.027848	*

Table S4 – Full results table for Multiple Linear Regression Model.
