# Supplementary material for: Key regulators control distinct transcriptional programmes in blood progenitor and mast cells
Source: EMBO J. 2014 Apr 23;33(11):1212–26. doi: 10.1002/embj.201386825 (PMC4168288; doi:10.1002/embj.201386825)
Supplement: Supplementary file 17 [file embj0033-1212-sd17.pdf]

**Model**	**Minimum # of TFs**	**# of genes tested**	**R^2 (adjusted)**	**REML**
without interaction	2	8261	0.346	19638
with interaction	2	8261	0.417	19422

Table S5 – Generalized Additive Model results.
