# Supplementary material for: Key regulators control distinct transcriptional programmes in blood progenitor and mast cells
Source: EMBO J. 2014 Apr 23;33(11):1212–26. doi: 10.1002/embj.201386825 (PMC4168288; doi:10.1002/embj.201386825)
Supplement: Supplementary file 19 [file embj0033-1212-sd19.pdf]

**Transcription factor**	**# of peaks - mast**
Mitf	4421
cFos	4798

Table S7 – Number of peaks in MITF and c-FOS ChIP-seq experiments.
