# Supplementary material for: Key regulators control distinct transcriptional programmes in blood progenitor and mast cells
Source: EMBO J. 2014 Apr 23;33(11):1212–26. doi: 10.1002/embj.201386825 (PMC4168288; doi:10.1002/embj.201386825)
Supplement: Supplementary file 20 [file embj0033-1212-sd20.pdf]

Model	Minimum # of TFs	# of genes tested	R^2	REML
without interaction	2	8967	0.309	21684
with interaction	2	8967	0.380	21471

Table S8 – Results of Generalized Additive Model including Mitf and c-Fos.
