# Supplementary material for: Key regulators control distinct transcriptional programmes in blood progenitor and mast cells
Source: EMBO J. 2014 Apr 23;33(11):1212–26. doi: 10.1002/embj.201386825 (PMC4168288; doi:10.1002/embj.201386825)
Supplement: Supplementary file 23 [file embj0033-1212-sd23.pdf]

THE EMBO JOURNAL

Manuscript EMBO-2013-86825

Key regulators control distinct transcriptional programs in blood progenitor and mast cells

Fernando J. Calero-Nieto, Felicia S. Ng, Nicola K. Wilson, Rebecca Hannah, Victoria Moignard, Ana I. Leal-Cervantes, Isabel Jimenez-Madrid, Evangelia Diamanti, Lorenz Wernisch, Berthold Göttgens

Corresponding author: Berthold Göttgens, University of Cambridge

Review timeline:

Submission date:	06 September 2013
Editorial Decision:	11 October 2013
Revision received:	27 February 2014
Editorial Decision:	20 March 2014
Accepted:	20 March 2014

Transaction Report:

(Note: With the exception of the correction of typographical or spelling errors that could be a source of ambiguity, letters and reports are not edited. The original formatting of letters and referee reports may not be reflected in this compilation.)

Editor: Thomas Schwarz-Romond

1st Editorial Decision 11 October 2013

Thank you very much for considering The EMBO Journal for presentation of your results. I am pleased to enclose significant, while at the same time very constructive comments from two expert scientists on the submitted dataset.

As you will see, three major criticisms arise from their remarks:

-ref#1 finds the paper much too descriptive and therefore demands much stronger support for the general functionality of TF's binding, thus experimentally establishing the favored functional versus opportunistic binding model.

-ref#2 very much echo's such functional concerns (please see his/her major point 2);
Reaching even further, ref#2 suggests to sharpen the posed problem while refining the (by than stronger functionally validated) major conclusions;

Conditioned on such rather critical amendments, I am happy to at least offer the change for a major round of amendments to the current manuscript. Please notice however that the referees, are currently indicating only medium novelty and general interest, an issue that will have to be

overcome by investing significant efforts to generalize the study and thoroughly integrating it into the context of published work.

Therefore, I urge you to consider whether you attempt revision for The EMBO Journal or alternatively may prefer to present the study rapidly elsewhere and without further delay. Please also note that a subsequent assessment will involve the original referees before reaching a final decision.

Please do not hesitate to get in touch regarding potential feasibility and/or a realistic timeline, in case you embark on revisions for our title (due to time constrains, preferably via E-mail).

I look forward to hear from you and remain with best regards.

REFEREE REPORTS:

Referee #1 (Report):

In the manuscript entitled "Key regulators control distinct transcriptional programs in blood progenitor and mast cells", the authors Calero-Nieto et al. claim that transcription factor binding (TF) is functional rather than opportunistic and contributes to cell-type specific transcriptional control. The authors performed ChIP-seq studies on ten TFs in hematopoietic progenitor and mast cell lines. The authors built an elegant mathematical model to correlate TF binding with changes in gene expression, and they examine how commonly expressed TFs contribute to the cell-type specific programs. Specifically, they show that knockdown of GATA2 and EGR in mast cells leads to deregulated expression of mast cell-specific genes. Finally, the authors find that known mast cell regulators, such as MITF and C-FOS, contribute to the global reorganization of TF binding profiles.

In general, the data of are convincing. However, the authors draw general conclusions from rather descriptive experiments. The authors present some functional data but fail to convincingly show that TF binding sites are generally functional. An inclusion of additional experiments, tempering of conclusions and discussion of published work in this area of research would improve this manuscript.

Specific comments:

1) The authors compare TF binding in different cell types, and they conclude that the cellular environment influences the global binding pattern of TF. However, it has been shown that "master regulators" are able to "open chromatin" and bind to target genomic regions independently of the cell environment (Cell 147:565-76, 2011; EMBO J. 31:4318-33, 2012). The authors should discuss their results in light of published work.

2) The authors claim that TF-specific motifs attract TFs to specific genomic regions. It is well established that TFs bind only to a fraction of available motifs (Nucleic Acids Res. 40 :5819-31, 2012). The authors should comment on these findings.

3) The authors perform a motif analysis and conclude that "binding of shared TFs to cell type specific regions is largely mediated through direct DNA binding via established motifs". The motif analysis does not exclude the involvement of protein-protein interactions in the recruitment or stabile binding of TFs. For example, it has been shown that knockdown of Oct4 in ES cells disrupts the binding of SMAD3 (Cell 147: 565-76, 2011). The authors should perform ChIP-PCR experiments in cells in which one TF is knocked down to examine whether the binding of neighboring TFs is affected. This experiment would clarify whether or not protein-protein interactions are dispensable for binding.

4) Using their mathematical model, the authors show that the maximum observed correlation of binding sites to gene expression changes, is 41.7%. This result implies that a great proportion of bound sites does not correlate with gene expression changes. In accordance with this finding, it has been shown that stability TF binding correlates with functionality, whereas instable TF binding reflects opportunistic binding (Nature 484: 251-5, 2012). The authors should explain how their findings correlate with this previously published work, and they should temper their main

conclusion.

5) The authors should include numbers and percentages in their motif and expression analysis to make the manuscript more understandable. For example, how many motifs are associated with each binding site? Do all factors bind constantly to the same motifs? How many sites do the factors occupy? How many of the bound genes correlate with gene expression changes?

6) Why did the authors study EGR and GATA2, especially since GATA2 has an already known role in mast cells? Since the authors claim that binding of TF is not opportunistic, they should present additional functional evidence for some of the other factors.

Minor points:

The authors should explain Supplementary Figure 2.

The authors interpret the motif analysis to suggest that Hox and bHLH factors play the role of "master regulators". The authors could perform overexpression and/or knockdown experiments to validate their claim.

The authors misrepresent the findings by Trompouki et al. (Cell 147: 577-589, 2011). This paper does not support an opportunistic model of binding but rather proposes that lineage-restricted regulators co-localize with signal-responsive TF and affect their binding. The authors should clarify this conclusion in their manuscript.

Referee #2 (Report):

This paper presents several important new RNA-seq and ChIP-seq datasets in a mouse model for hematopoietic stem-progenitor cells (the HPC7 cell line) and in cells from one of the mature progeny, i.e. cultured mast cells. The authors show that ten hematopoietic transcription factors (TFs) are present in both the HPC7 and mast cells, but the profiles of occupancy genome-wide are largely distinct to each cell type, as are the expression levels of a subset of genes. They present a combined approach of experiments and mathematical modeling to glean insights from these important new data. The explanatory power from the mathematical models is impressive (R-squared values over 0.4). However, the major conclusions need to be refined, especially with regard to "opportunistic" binding by TFs versus "functional" binding. For the most part, the data are strong and the presentation of Results is clear, but additional points below will help clarify a few issues.

1. The authors set up a major contrast between "opportunistic" binding by TFs and "functional" binding. They are correct that this and related issues are frequently debated in the context of the large number of binding sites observed in whole-genome mapping of TF occupancy, e.g. by ChIP-seq. First, they need to define the contrast in consistent terms. In this version of the manuscript, they seem to consider "opportunistic" binding as resulting from TFs being directed to binding sites by the cellular environment, such as accessible chromatin. They do not directly define "functional", but they should. For instance it could mean binding that generates a measurable effect in gain of function or loss of function assays. However, to distinguish between opportunistic and functional, they should be defined as opposites or at least non-overlapping concepts. Functional binding (e.g. shown by experimental intervention to be needed for enhancement or promotion) can occur at locations accessible for binding, as evidenced by DNase sensitivity and appropriate histone modifications that exist prior to binding by the TF. I'm sure the authors are aware of such examples, and it is important to incorporate this into the way they set up the contrast.

2. The authors list three or four results (Abstract, Discussion) that they interpret as supporting functionality of binding, in contrast to "opportunistic" binding, but these results are not compelling for this contrast. The fact that their mathematical models have good predictive power shows that some of the bound sites are contributing to function, but it need not be a majority (see points 3, 7); this observation is still consistent with "opportunistic" binding at some (many?) locations. The fact that expected TF binding site motifs are enriched in the TF-bound DNA segments does not

necessarily imply function at a majority of the sites. Knock-down experiments do show a role for TFs at particular targets, but again this does not rule out a substantial amount of "opportunistic" binding. These are all important observations, but they do not address the (still vague) distinction in a compelling manner.

3. Given points 1 and 2, the authors should consider reframing their central questions. One approach could consider the entire set of TF-bound segments as being either functional or not, and then estimating the fraction in each category, based on their mathematical modeling. That would generate an interesting, and possibly provocative, answer to the question of "What proportion of the TF-bound DNA segments are functional?" For the TF-bound segments that were examined in the modeling, the coefficients learned by training the regression models or GAMs could point to such an estimate. That is an example of an approach that would give concrete answers to clearly defined questions. Currently, questions such as that posed on line 179 (what is the "extent to which cell type-specific binding of shared TFs might be associated with gene expression"?) are not answered.

4. The claims on page 10 need to explained and possibly toned-down. The authors say "Predictions obtained using GAM were more accurate than the linear regression model even in the absence of interaction terms." However they get R-squared of 41.4% for linear regression (with thresholding) vs 41.7% for GAM with interactions. Surely this is not a significant difference. Similarly, the authors should be cautious about their statement in the Discussion that "GAM more than doubled the R-squared values." Exactly what is being compared?

5. The authors conduct an interesting analysis of motif occurrences in the TF-bound segments. This does point to DNA sequences directing binding at a sufficiently large number of sites for the motif to be enriched. However, it does not rule out a substantial amount of binding by "tethering of shared factors to regulatory elements through protein-protein interaction with cell type specific factors". Both could be going on, perhaps at distinct subsets of binding locations.

6. The authors make an important point about recursively generating experimental data, doing modeling, and then doing additional experiments based on the modeling results, etc. They have a great opportunity here that seems to be missed. Having performed additional ChIP-seq on MITF and C-FOS, based on results from their modeling, how much improvement in the modeling results occurs when these data are incorporated? The current description of the "improvement" (page 12) is hard to interpret, having to do with the number of binding events for MITF and C-FOS (one versus more than one).

7. The authors should provide more information about their mathematical modeling:
(a) How many (or what proportion of) TF-bound segments were included in the modeling? They had to be within 50 kb of a gene.
(b) What was the rationale for simply averaging all the delta_TF coverages for all TF-bound segments assigned to a gene? Couldn't a subset of them be playing a major role?
(c) Results from multiple linear regression models were averaged into one R-squared value. How much variation is there in R-squared among the models?
(d) Generalized Additive Models (GAMs) were trained and tested only for the genes with more than one TF bound. How many genes and TF OSs were included?

8. The knock-down and promoter mutation experiments (p. 13) do not address issues essential to the main theme of the paper. These experiments are good tests of hypotheses derived from the earlier genetic experiments showing a role for GATA2 in mast cells. If there is something from the new genome-wide data and modeling that leads to these experiments, it should be stated explicitly.

9. The claim of priority for "comprehensive computational and experimental analysis illustrating how multiple key regulatory TFs contribute to transcriptional programs in distance mammalian cell types" (p. 5) is unnecessary and hard to prove. Certainly several comprehensive studies about the role of multiple key TFs in ES cells and other hematopoietic cells have been published (some from this group), and each has a substantial computational and experimental component.

10. Standard names for genes and proteins should be used throughout. For instance, mouse proteins are all uppercase.

11. p. 21, line 484: "genes" should be "peaks"

12. p. 22, line 510: What is "REML estimation"? This should be referenced.

13. line 59: TF binding in mammalian repeats was reported earlier, by Guilliam Bourque et al. (2008, Genome Res. 18:1752-1762)

14. Purity (homogeneity) of the mast cells after culture should be stated, perhaps with a FACS analysis in the Supplement.

1st Revision - authors' response 27 February 2014

Referee #1 (Report):

We were pleased to read this reviewer's conclusion that "*in general, the data are convincing*". The referee also commented that "*inclusion of additional experiments, tempering of conclusions and discussion of published work in this area of research would improve this manuscript*". To achieve this, the referee made a number of very constructive comments, which we have addressed in the revised manuscript as outlined below:

Specific comments:

1) The authors compare TF binding in different cell types, and they conclude that the cellular environment influences the global binding pattern of TF. However, it has been shown that "master regulators" are able to "open chromatin" and bind to target genomic regions independently of the cell environment (Cell 147:565-76, 2011; EMBO J. 31:4318-33, 2012). The authors should discuss their results in light of published work.

We are grateful to the referee for raising this valid point, and have added a new section to the discussion as suggested (page 19 of revised manuscript).

2) The authors claim that TF-specific motifs attract TFs to specific genomic regions. It is well established that TFs bind only to a fraction of available motifs (Nucleic Acids Res. 40 :5819-31, 2012). The authors should comment on these findings.

We completely agree with the referee's point that only a fraction of available motifs in the genome are bound by TFs, which indeed represents one of the big unsolved mysteries in transcription factor function. The point we were trying to make is that when TFs bind to different sets of regions in 2 different cell types, we observed that their consensus binding motifs were statistically enriched in both sets of sequences. We have commented on this in page 19 of revised manuscript.

3) The authors perform a motif analysis and conclude that "binding of shared TFs to cell type specific regions is largely mediated through direct DNA binding via established motifs". The motif analysis does not exclude the involvement of protein-protein interactions in the recruitment or stable binding of TFs. For example, it has been shown that knockdown of Oct4 in ES cells disrupts the binding of SMAD3 (Cell 147: 565-76, 2011). The authors should perform ChIP-PCR experiments in cells in which one TF is knocked down to examine whether the binding of neighbouring TFs is affected. This experiment would clarify whether or not protein-protein interactions are dispensable for binding.

We agree with the reviewer that overrepresentation of motifs does not exclude a possible role for protein-protein interactions in recruitment of stable TF binding at a specific regulatory region.

When considering the whole set of regions however, motif overrepresentation does provide strong statistical evidence that TF interactions with their cognate motifs play an important role. To provide further statistical evidence, we have now calculated for each set of TF-bound regions the percentage of target regions that contained the relevant consensus motif for each factor (Figure 4 of revised manuscript). This new analysis shows that the fraction of regions with consensus motif differs for different TFs, and as suggested by the reviewer, implicates indirect binding / recruitment for a significant fraction of the TF binding events we have observed.

Notwithstanding this additional computational analysis, we do also completely agree with the reviewer that additional experimental data can help to clarify this issue. We have therefore performed the ChIP-PCR experiments following TF knock-down, as suggested by the reviewer. Specifically, we performed knock-down of Fli1, Pu.1 and Gata2 followed by ChIP for those 3 factors, and we analysed the consequences on TF-binding at 3 different loci, including Cx3cr1, which was analysed by mutagenesis experiments in the original submission. These new experiments showed that recruitment of ETS factors depended on the presence of consensus motifs and seemed to be unaffected by the reduction of Gata2. Gata2 was dependant on the presence of its consensus motif although recruitment of the ETS factor Fli1 was also important. Our results therefore clearly show the importance of both binding through established consensus motifs as well as presumed tethering by other TFs (also see response to point 5 below). We have modified Figure 6 and the text on page 15 and added a new figure (Figure E11) to include these important new results.

4) Using their mathematical model, the authors show that the maximum observed correlation of binding sites to gene expression changes, is 41.7%. This result implies that a great proportion of bound sites does not correlate with gene expression changes. In accordance with this finding, it has been shown that stability TF binding correlates with functionality, whereas instable TF binding reflects opportunistic binding (Nature 484: 251-5, 2012). The authors should explain how their findings correlate with this previously published work, and they should temper their main conclusion.

Upon re-reading the text, we realize now that we may not have provided the best explanations to interpreting the model results and there seems to be a misunderstanding about what the value 0.417 mentioned above corresponds to. To address this, we have amended the text in the results and discussion sections (see pages 9, 10 and 17 of revised manuscript). As pointed out, the GAM model with TF interactions gave an R^2 value of 0.417 indicating that 41.7% of gene expression variation can be explained by changes in the 10 shared TFs. This corresponds to a correlation $R = 0.65$ between observed and predicted values of the model. In the multiple linear regression models, the relationship between changes in TF binding and gene expression is defined by the coefficients of the predictor variables (see Tables EIV and EVI of revised manuscript) whereas in the GAM model, the smoothing functions describe a non-linear function that relates each TF binding to gene expression.

In the article mentioned by the referee, sites of stable binding were strongly correlated with sites of enrichment for histone acetyltransferases, including those for acetylation of lysine 27 of histone H3 (H3AcK27). For this revised version of the manuscript, we have therefore now performed additional ChIP-Seq experiments for this histone mark in HPC7 and mast cells, and have analysed levels of this histone mark in relation to the binding events for the 10 TFs. We found that TF-bound regions that performed well in our mathematical model were associated with high levels of H3K27 Acetylation. We have added a figure (Figure E6) and a new section in the text (page 13 of revised manuscript) to show this, and also have amended the text in line with the reviewer's suggestions.

5) The authors should include numbers and percentages in their motif and expression analysis to make the manuscript more understandable. For example, how many motifs are associated with each binding site? Do all factors bind constantly to the same motifs? How many sites do the factors occupy? How many of the bound genes correlate with gene expression changes?

We appreciate the reviewer's suggestions for incorporating more quantitative data on motif and expression analysis, and have therefore performed substantial new analysis, which we have incorporated into the revised Figures E2A, 4, E6 and E7B. Figure E2A was added to show the

repertoire of TF bound genes and the associated gene expression levels in HPC7 and mast cells. 58.6% of genes expressed in either cell type are bound by at least 1 shared TF. These genes show similar distribution of expression scores as the entire transcriptome shown in Figure 1.

For the revised figure 4, we screened all the bound sites for each motif and calculated the percentage based on the total number of sites for each category. We illustrate these results by amending the heatmap to show these percentage values. When we compare the pattern of motif enrichment across the 3 categories (HPC7-specific, common and mast-specific), motifs of shared TFs were similarly enriched between the categories suggesting that shared TFs were recruited to these regions and bind DNA without preference for cell-type specific or common regions. This is in contrast to a recent finding by Gertz et al. (2013. Mol Cell, 52: 25-36), where the authors showed that estrogen receptor cell-type-specific regions lack its binding motif, ERE (estrogen response elements), while shared regions have strong enrichment of ERE. The authors proposed a model where ER is tethered to co-factors at cell-type-specific sites but binds directly to DNA at shared sites. By contrast, our data suggest that for the factors investigated here, both direct and tethered binding occur, regardless of whether binding events are cell-type specific or shared.

In Figure E6 we addressed the association of good prediction capabilities by our mathematical model with high levels of H3K27 acetylation (see previous point).

Figure E7B shows the fraction of differentially expressed genes following TF knock-down that contained binding peaks in their loci for the respective TF. This fraction ranged from 10.8 to 70.3%.

We discuss these new observations in the revised manuscript.

6) Why did the authors study EGR and GATA2, especially since GATA2 has an already known role in mast cells? Since the authors claim that binding of TF is not opportunistic, they should present additional functional evidence for some of the other factors.

We are grateful to the referee for raising this valid point. To answer the referee's question, we have performed additional know-down experiments in primary mast cells followed by analysis of expression by microarrays. We have analysed the role of 2 more ETS factors (Fli1 and Pu.1), 1 bHLH factor (E2a) and Lmo2 and we could see that all these shared TFs make significant contributions to gene expression control in mast cells. These new results are shown in the new figures E7 and E8. To analyse the importance of those factors for mast cell growth, we have also now performed growth competition experiments in the mast cell line MST between uninfected cells and those where one of the shared TF was knocked-down. Results showed that TF knock-down lead to a growth disadvantage, with particularly striking results for Fli1 and Gata2. These new results are shown in figure E9, and discussed in the text on page 14 of revised manuscript.

Minor points:

1) The authors should explain Supplementary Figure 2.

Legend for Supplementary Figure 2 (Figure E2B in revised manuscript) has been amended.

2) The authors interpret the motif analysis to suggest that Hox and bHLH factors play the role of "master regulators". The authors could perform overexpression and/or knockdown experiments to validate their claim.

As this was listed as a minor point, we felt it was appropriate to address this issue by amending the text of our paper. Especially since it is known already that the bHLH factor Mitf is a major regulator of mast cell fate, and the same is true for Hox factors with respect to the blood stem/progenitor fate. We have therefore amended the text (page 11 of revised manuscript) to highlight this point.

3) The authors misrepresent the findings by Trompouki et al. (Cell 147: 577-589, 2011). This paper does not support an opportunistic model of binding but rather proposes that lineage-restricted regulators co-localize with signal-responsive TF and affect their binding. The authors should clarify this conclusion in their manuscript.

We have taken this comment on board and revised the discussion (see page 19 of revised manuscript).

Referee #2 (Report):

We were delighted to read the many positive comments by this reviewer including "*this paper presents several important new RNA-seq and ChIP-seq datasets in a mouse model for hematopoietic stem-progenitor cells (the HPC7 cell line) and in cells from one of the mature progeny, i.e. cultured mast cells", "the explanatory power from the mathematical models is impressive (R-squared values over 0.4)",* and *"for the most part, the data are strong and the presentation of results is clear".*

The reviewer also commented that "*the major conclusions need to be refined, especially with regard to 'opportunistic' binding by TFs versus 'functional' binding*", and he/she provided a number of very constructive points to clarify these issues, which we have addressed as outlined below:

1. The authors set up a major contrast between "opportunistic" binding by TFs and "functional" binding. They are correct that this and related issues are frequently debated in the context of the large number of binding sites observed in whole-genome mapping of TF occupancy, e.g. by ChIP-seq. First, they need to define the contrast in consistent terms. In this version of the manuscript, they seem to consider "opportunistic" binding as resulting from TFs being directed to binding sites by the cellular environment, such as accessible chromatin. They do not directly define "functional", but they should. For instance it could mean binding that generates a measurable effect in gain of function or loss of function assays. However, to distinguish between opportunistic and functional, they should be defined as opposites or at least non-overlapping concepts. Functional binding (e.g. shown by experimental intervention to be needed for enhancement or promotion) can occur at locations accessible for binding, as evidenced by DNase sensitivity and appropriate histone modifications that exist prior to binding by the TF. I'm sure the authors are aware of such examples, and it is important to incorporate this into the way they set up the contrast.

We are very grateful for this constructive comment, and completely agree with the reviewer that our paper can be improved by inclusion of a more precise definition of the concepts of opportunistic v. functional TF binding. We have therefore amended the text of the introduction (line 68 of revised manuscript) taking on board the very welcome suggestions provided by the reviewer.

2. The authors list three or four results (Abstract, Discussion) that they interpret as supporting functionality of binding, in contrast to "opportunistic" binding, but these results are not compelling for this contrast. The fact that their mathematical models have good predictive power shows that some of the bound sites are contributing to function, but it need not be a majority (see points 3, 7); this observation is still consistent with "opportunistic" binding at some (many?) locations. The fact that expected TF binding site motifs are enriched in the TF-bound DNA segments does not necessarily imply function at a majority of the sites. Knock-down experiments do show a role for TFs at particular targets, but again this does not rule out a substantial amount of "opportunistic" binding. These are all important observations, but they do not address the (still vague) distinction in a compelling manner.

These comments all provided real food for thought, and were therefore very much appreciated. We have taken them on board in response to the specific points below.

3. Given points 1 and 2, the authors should consider reframing their central questions. One approach could consider the entire set of TF-bound segments as being either functional or not, and then estimating the fraction in each category, based on their mathematical modelling. That would generate an interesting, and possibly provocative, answer to the question of "What proportion of the TF-bound DNA segments are functional?" For the TF-bound segments that were examined in the modelling, the coefficients learned by training the regression models or GAMs could point to such an estimate. That is an example of an approach that would give concrete answers to clearly defined questions. Currently, questions such as that posed on line 179 (what is the "extent to which cell type-specific binding of shared TFs might be associated with gene expression"?) are not answered.

As mentioned in comment 2, we have included a more precise definition of the term "functional": those binding events that are relevant in terms of transcriptional control processes. It is well established that acetylation of lysine 27 of histone H3 (H3AcK27) is a mark of active regions in the genome (Creyghton et al. 2010. PNAS, 107: 21931-21936). For this revised version of the manuscript, we have therefore performed ChIP-Seq for this histone mark in HPC7 and mast cells and have compared enrichment levels with the binding events of the 10 TFs. We found that TF-bound regions that performed well in our mathematical model were associated with high levels of H3K27 Acetylation. We have added a new figure (Figure E6) to show this, and also have amended the text to state that, while there is still much that we do not understand, our model captures biologically relevant links between differential TF binding and differential gene expression, that shows a significant overlap with other known features of active gene regulatory elements, such as H3K27 acetylation (page 13 of revised manuscript).

4. The claims on page 10 need to explained and possibly toned-down. The authors say "Predictions obtained using GAM were more accurate than the linear regression model even in the absence of interaction terms." However they get R-squared of 41.4% for linear regression (with thresholding) vs 41.7% for GAM with interactions. Surely this is not a significant difference. Similarly, the authors should be cautious about their statement in the Discussion that "GAM more than doubled the R-squared values." Exactly what is being compared?

We now recognise that our original statements were confusing. The value 0.414 corresponds to predictions obtained with the linear regression model using only those genes that have TF-bound regions that are simultaneously bound by 5 or more TFs, which corresponds to only 1223 genes. However, the value 0.417 obtained for the GAM model corresponds to predictions using all genes that have TF-bound regions that are simultaneously bound by 2 or more TFs, which corresponds to 8261 genes. The R-squared value for this much larger gene set is only 0.252 in the linear regression model. We have amended the text in order to clarify this point and we have modified our statements accordingly (see pages 10 and 17 of revised manuscript).

5. The authors conduct an interesting analysis of motif occurrences in the TF-bound segments. This does point to DNA sequences directing binding at a sufficiently large number of sites for the motif to be enriched. However, it does not rule out a substantial amount of binding by "tethering of shared factors to regulatory elements through protein-protein interaction with cell type specific factors". Both could be going on, perhaps at distinct subsets of binding locations.

We fully agree with the referee that direct as well as tethered binding are likely to occur at different locations. We have therefore performed additional calculations that have allowed us to modify figure 4 to indicate the proportion of DNA fragments that contain the relevant motifs. From this new figure, it is clear that recruitment by protein-protein interactions likely takes place for a significant proportion of the binding events we have observed in this study. However, as already recognised by

the referee, direct binding in our dataset is frequent enough to be able to discover the motifs by de novo motif discovery. Moreover, we showed that mutation of motifs implicated in direct binding has profound effects on transcriptional activity. The situation therefore appears to be exactly as suggested by the reviewer, i.e. a mixture of direct and indirect binding mechanisms.

To explore this issue further, we also performed knock-down of three factors followed by ChIP for the same three factors (Fli1, Pu.1 and Gata2). Quantitative analysis of binding at different loci was indeed consistent with the occurrence of both direct and indirect binding mechanisms (see new Figures 6D and E11 and relevant text on page 15 of revised manuscript; also see response to point 3 by referee 1).

6. The authors make an important point about recursively generating experimental data, doing modelling, and then doing additional experiments based on the modelling results, etc. They have a great opportunity here that seems to be missed. Having performed additional ChIP-seq on MITF and C-FOS, based on results from their modelling, how much improvement in the modelling results occurs when these data are incorporated? The current description of the "improvement" (page 12) is hard to interpret, having to do with the number of binding events for MITF and C-FOS (one versus more than one).

Following up on this suggestion, we have now included additional modelling results where Mitf and c-Fos are considered. Since Mitf is not expressed in HPC7 cells and c-Fos expression is so very low that ChIPs do not give specific enrichment, we utilized the IgG ChIP-Seq to represent background levels of these 2 TFs in HPC7 so that differential binding can be quantified between mast and HPC7 cells. Although the resulting GAM model including 12 TFs did not show an increase in the R^2 values, inclusion of the two extra factors resulted in the inclusion of ~700 more genes. The new model therefore explains similar amounts of variation (as the 10TF model) but on a larger set of genes. We have included a new figure (Figure E4), 2 new Tables (EVIII and EIX) and added a new paragraph in the manuscript (page 13 of revised manuscript) to outline these findings.

7. The authors should provide more information about their mathematical modelling:

(a) How many (or what proportion of) TF-bound segments were included in the modelling? They had to be within 50 kb of a gene.

There were 151,925 TF bound regions in total. 110,667 regions mapped to 19,163 genes and 41258 regions did not map to any genes. Of 110,667 regions mapped to genes, 46,536 regions were bound by 2 or more TFs. We now provide this information in the methods section (page 23 of revised manuscript).

(b) What was the rationale for simply averaging all the delta_TF coverages for all TF-bound segments assigned to a gene? Couldn't a subset of them be playing a major role?

We agree with the reviewer that single elements could play a major role, and that such effects may, to some extent, be diluted by averaging out across the gene locus. To account for such effects however would require detailed knowledge of all gene loci, as they would have to be assessed on a case-by-case basis. Importantly, our model is designed to capture expression levels, where the expression level of a given gene serves as a single output function integrating the inputs from the various regulatory elements. To assign a unique value to this single output function, our options were to consider all regions or a subset of them. A subset might have included several regions matching a given set of criteria or just the one region with the maximum value. We discarded the latter option since it is widely accepted that genes are commonly under the control of several regulatory regions, all looping to the promoter. If we had only considered a subset of regions, this would have involved setting a threshold, but we felt that assigning a unique and consistent threshold along the entire dataset would be difficult without introducing a bias. Therefore, to consider all regions seemed the best option.

We also explored the utility of weighting according to distance to the transcription start site, as this has been used in the past (Ouyang, et al. PNAS, 2009. 106(51): p. 21521-6). This however did not improve the performance of our model, which we interpreted as a manifestation of the long –

© EMBO

recognised feature of DNA looping, which implies that distance along the linear DNA sequence is not necessarily related to physical distance inside the nucleus.

(c) Results from multiple linear regression models were averaged into one R-squared value. How much variation is there in R-squared among the models?

The variation among the models is indicated by the standard error bars in the bar chart of Figure 3B. We have amended the figure legend to highlight this important point.

(d) Generalized Additive Models (GAMs) were trained and tested only for the genes with more than one TF bound. How many genes and TF OSs were included?

As indicated in the Material and methods (line 557 of revised manuscript), only genes with more than 2 TF bound were used for the GAM model (this allowed us to consider pairwise interactions). A total of 8261 genes and 10 TFs were included in the model as indicated in Table EV.

8. The knock-down and promoter mutation experiments (p. 13) do not address issues essential to the main theme of the paper. These experiments are good tests of hypotheses derived from the earlier genetic experiments showing a role for GATA2 in mast cells. If there is something from the new genome-wide data and modelling that leads to these experiments, it should be stated explicitly.

Please refer to point 6 of referee 1.

9. The claim of priority for "comprehensive computational and experimental analysis illustrating how multiple key regulatory TFs contribute to transcriptional programs in distance mammalian cell types" (p. 5) is unnecessary and hard to prove. Certainly several comprehensive studies about the role of multiple key TFs in ES cells and other hematopoietic cells have been published (some from this group), and each has a substantial computational and experimental component.

We have taken this on board and amended the text accordingly (line 97 of revised manuscript).

10. Standard names for genes and proteins should be used throughout. For instance, mouse proteins are all uppercase.

The text has been amended accordingly.

11. p. 21, line 484: "genes" should be "peaks"

The text has been amended (line 530 of revised manuscript).

12. p. 22, line 510: What is "REML estimation"? This should be referenced.

REML stands for Restricted maximum likelihood. We have amended the text and added the reference (line 560 of revised manuscript). (Wood SN (2011), J R Stat Soc B]

13. line 59: TF binding in mammalian repeats was reported earlier, by Guilliam Bourque et al. (2008, Genome Res. 18:1752-1762)

Reference has been changed (line 58 or revised manuscript).

14. Purity (homogeneity) of the mast cells after culture should be stated, perhaps with a FACS analysis in the Supplement.

A new figure has been added to include the purity of mast cells (Figure E12), and we now refer to this figure in the methods (page 20 of revised manuscript) to direct the reader to this information.

2nd Editorial Decision 20 March 2014

Thank you very much for the revised study.

One of the original referees assessed your revised paper with the compliments being enclosed below.

I am thus very happy to proceed with formal acceptance/publication and take the liberty to congratulate at this point to such a fine study.

To enable rapid proceedings, I would be delighted to receive:

- A 2 up to 4 'bullet point' synopsis, that summarizes the major novelty/advance provided by your study.

- We have the opportunity to graphically highlight selected studies at our new 'image carousel' at our homepage (http://emboj.embopress.org).

This applies to a limited number of published papers only and I would be delighted if you could provide an integrating figure (graphical abstract) in the format of 550 x 150 (up to 400) pixel for this purpose.

REFEREE REPORT:

The authors have addressed all my concerns thoroughly, both with changes to the text and new experimental data. This paper describes a strong advance not only in our knowledge of hematopoiesis and differentiation to mast cells, but more generally for our understanding of gene regulation. It illustrates how the cycle of gathering high quality, comprehensive data on expression and TF binding, followed by mathematical modeling, and then testing predictions derived from that modeling leads to important new insights. Furthermore, they develop a solid case for much of the TF binding being functional rather than opportunistic.
